# Trees, Coral, and Seaweed: An Interpretation of Sketches Found in Darwin’s Papers

**DOI:** 10.1007/s10739-019-09591-4

**Published:** 2020-02-04

**Authors:** Kees van Putten

**Affiliations:** 1 Faculty of Philosophy, Theology and Religious Studies, Radboud University, Houtlaan 4, 6525 XZ Nijmegen, The Netherlands; 2 Snoeksloot 14, 3993HL Houten, The Netherlands

**Keywords:** Darwin’s sketches, Darwin’s heuristics, Graphic elements in Darwin’s diagram and sketches, Evolutionary descent, Phylogenetic trees, Darwin’s tree diagram

## Abstract

The sole diagram in *On the Origin of Species* is generally considered to be merely an illustration of Darwin’s ideas, but such an interpretation ignores the fact that Darwin himself expressly stated that the diagram helped him to discover and express his ideas. This article demonstrates that developing the so-called “tree diagram” substantially aided Darwin’s heuristics. This demonstration is based on an interpretation of the diagram and of 17 sketches found in Darwin’s scientific papers. The key to this interpretation is the meaning that Darwin assigned to the graphic elements (points, lines, and spaces) he used to construct the preliminary sketches and the diagram. I argue that each of the sketches contributed to the shaping of Darwin’s ideas and that, in their succession, each added new elements that ultimately resulted in the fully developed published diagram.

## Introduction: Seventeen Sketches and a Diagram

Darwin’s collected papers contain 17 sketches executed before 1859, each one standing in a particular relation to the single diagram that appears in *On the Origin of Species* (1859).^1^ It was not until the beginning of the twenty-first century that these drawings attracted careful attention of scholars interested in how Darwin formed his ideas, most prominently Julia Voss, Horst Bredekamp, J. David Archibald, and Heather Brink-Roby.

Voss relates the sketches to diagrams of the “natural system” published by Darwin’s predecessors and contemporaries and to the ideas represented in those diagrams (2010). Bredekamp characterizes Darwin’s drawings as analogies, metaphorical images, models, visual thought experiments, and bearers of Darwin’s thinking process (2006, pp. 18–27, 34–59). He concludes that the diagram in *Origin* has the structure not of a tree (as often described) but of a coral, and that the same goes for all of Darwin’s sketches that precede the diagram. Archibald calls Darwin’s drawings “visual metaphors.” Like Voss and Bredekamp, he positions the sketches in the age-old tradition (common among naturalists before and after Darwin) of representing nature’s order by ladder- or tree-like images (Archibald 2014, pp. 80–113). My position, which I will present in the following pages, differs from these authors.^2^

Unlike the ladders or trees of nature that were drawn by other naturalists, Darwin’s sketches were not meant to be published. Therefore, they must have had a personal function. Above one of his sketches, for example, he wrote: “a tree not good simile—endless piece of seaweed dividing” (see below Fig. 7). It is clear that at the time he drew this specific sketch, he was searching for a proper simile. In other words, he strove to achieve maximal similarity between his sketch and the theme of the sketch: the affinities between organisms. This also follows from a passage in *Origin* that introduces the book’s sole figure, a “diagram”:

As it is difficult to show the blood-relationship between the numerous kindred of any ancient and noble family, even by the aid of a genealogical tree, and almost impossible to do this without this aid, we can understand the extraordinary difficulty which naturalists have experienced in describing, without the aid of a diagram, the various affinities which they perceive between the many living and extinct members of the same great natural class. (Darwin 1859, p. 431)

These quotations indicate that when he executed each one of his sketches, Darwin sought for the greatest similarity between them and the ways in which he conceptualized the mutual affinities between living species as well as extinct ones.

**Fig. 1:**
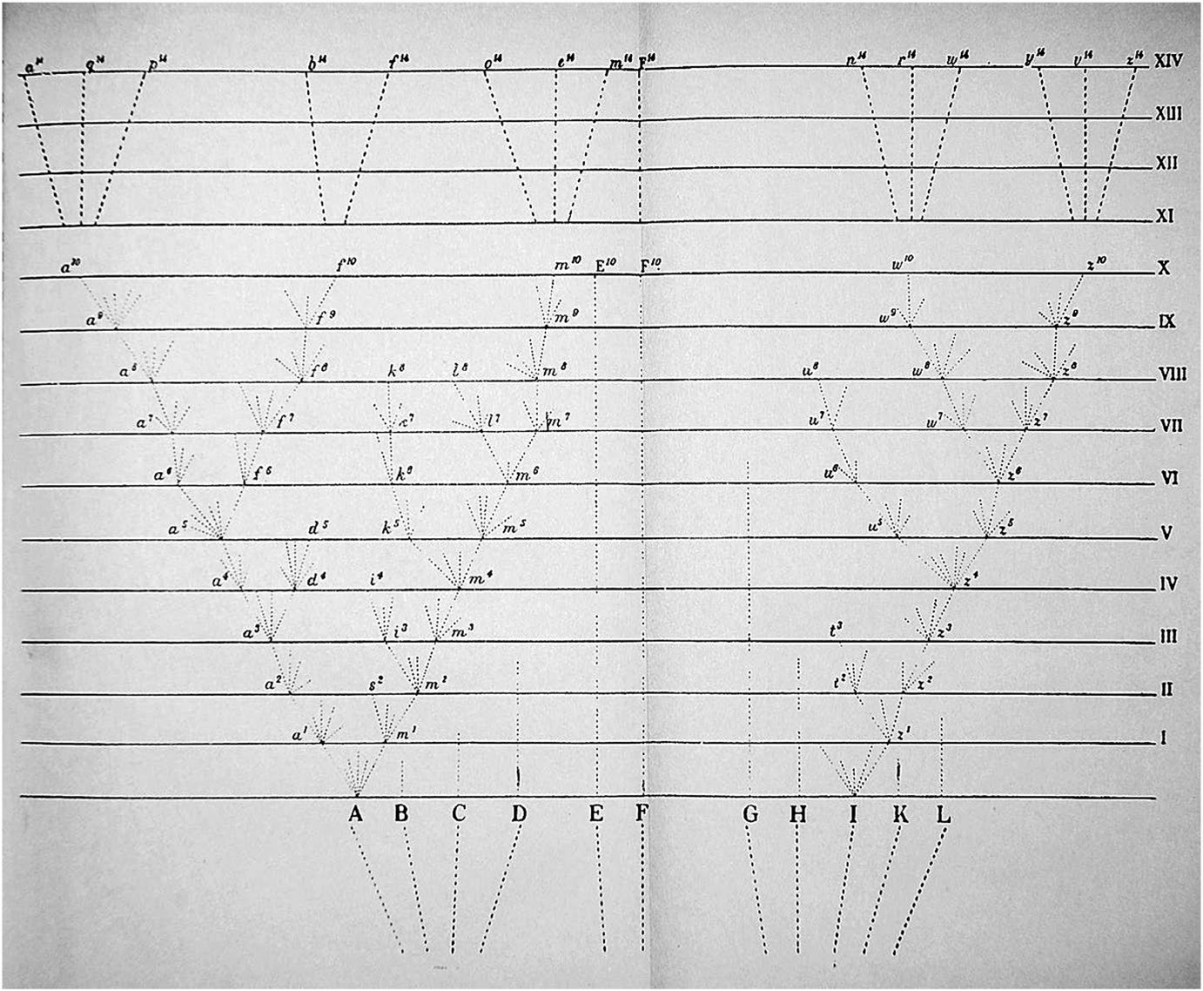
The sole figure in *The Origin*—in the original edition, a loose centerfold inserted between pp. 116–117. (Reproduced from Wiki commons: https://commons.wikimedia.org/wiki/File:Darwin_divergence.jpg, accessed 12 May 2015.)

Darwin’s sketches were indispensable for shaping his ideas; that is, the sketches had a heuristic function. In his earliest sketches, the similarity depended on a concrete likeness to trees, corals, or seaweed, but over time, the sketches became more abstract diagrams. The published diagram in *Origin*, however, illustrates that Darwin consciously sought to depict the most accurate visual representation of his ideas (see Fig. 1). These two processes—developing one’s ideas by using images and seeking the optimal visualization to submit these ideas to the reader—are somewhat different tasks, and they occur at different moments in the course of discovery and dissemination.

In this paper, I aim to demonstrate that the sketches, regarded as a series and placed in chronological order, can be shown to have contributed to Darwin’s thought, and that they ultimately culminated in the diagram in *Origin.* In the diagram, elements originating from the sketches performed a didactic function, explaining to the reader what they had taught Darwin earlier. I will do this in two ways: first, by analyzing the sketches, zooming in on the functioning of their elements (points, lines, and spaces), and relating them to our additional knowledge about the development of Darwin’s thought on evolution, and second, by tracking elements of the sketches and their functioning in the diagram in *Origin*.

In addition, to complete the picture as much as possible, I will also relate Darwin’s sketches to the (limited) prehistory of visualizing evolutionary theories and its context. For example, prior to Darwin’s prolonged, sketch-supported thought process, Jean-Baptiste Lamarck published the first known evolutionary tree (1809, p. 643). In the 1840s, after Darwin had already drawn his first four sketches, Robert Chambers included a tree-like diagram in his *Vestiges of the Natural History of Creation* to demonstrate how embryological changes could be interpreted in terms of an evolutionary history (1844, p. 212). Below, in “In Search of an Evolutionary Principle of Descent and Extinction (1837)” and ““The Embryo is the Animal in Its Less Modified State” (1852–1855)” sections, I will relate Darwin’s sketches to these figures and show that Darwin truly did revolutionize the mode of visualization of evolution.^3^

## A Multifunctional Diagram (1859)

Darwin’s introduction to his diagram, quoted above, refers to the indispensable role that it plays in his work. He concludes that without a diagrammatic aid, it would have been nearly impossible to figure out how evolution simultaneously shapes nature and its taxonomy: “I believe this element of descent is the hidden bond of connexion which naturalists have sought under the term of the Natural System” (Darwin 1859, p. 433).

According to Darwin, *Origin* is actually “one long argument,” and the crux of this argument is Chapter 4 on “Natural Selection.” Having reached this point in his line of reasoning, Darwin needed to show how the principles of variation, natural selection, and heredity of characteristics combine in the dynamics of the evolutionary process. It is to that end that he introduced the diagram (see Fig. 1).

The compactness of the diagram’s explanatory power is inversely proportional to the length of the text referring to it. In order to help the reader understand all that the diagram demonstrates, Darwin included a comprehensive and detailed explanation in four parts, which appears in various places in the text and takes up a total of sixteen pages. First of all, he presents the elements of his theory: variation; competition; divergence of characteristics; selection; the way variations establish themselves in niches in the “Economy of Nature”; extinction; the explosive growth of species in nature; the incompleteness of the “geological archive”; similarities and differences within and between races, species, and genera; and the absolute necessity that biological taxonomies match evolutionary reality in nature. He also maintains in the first part of his explanation that the diagram simultaneously provides the details and an overview of his theory and that it reveals—also simultaneously—a drawn history as well as specific moments in that history (along with the present, for anyone willing to see it).

The diagram manages to show all of this because it combines graphic and typographic elements, to which Darwin has assigned interrelated meanings. The graphic elements are *points* and *lines* of various kinds. The typographic elements are *letters*, *numerals,* and—least visible, but most revealing—horizontal *spaces* of various lengths. Using these five elements, Darwin illustrates how evolution works in nature.

The points labeled with capital letters on the horizontal lines at the bottom designate the original moments in which variation begins to lead to divergence of characteristics, which in the end brings about speciation. Each of the points designated by lowercase letters in the climbing lines symbolize an abstractly large number of morphing new varieties. The horizontal typographic spaces symbolize the measure of divergence: the wider the spacing, the greater the divergence. Clearly, these elements of the diagram function as bearers of meaning. Likewise, Darwin’s sketches also consist of meaning-bearing points, lines, and spaces. It is my contention that Darwin gained experience over time in assigning meaning to the graphic elements of his sketches, and that historians can interpret the diagram in *Origin* as the culmination of this long process of diagrammatic thinking. In the following sections I will reconstruct this process.

## In Search of an Evolutionary Principle of Descent and Extinction (1837)

**Fig. 2:**
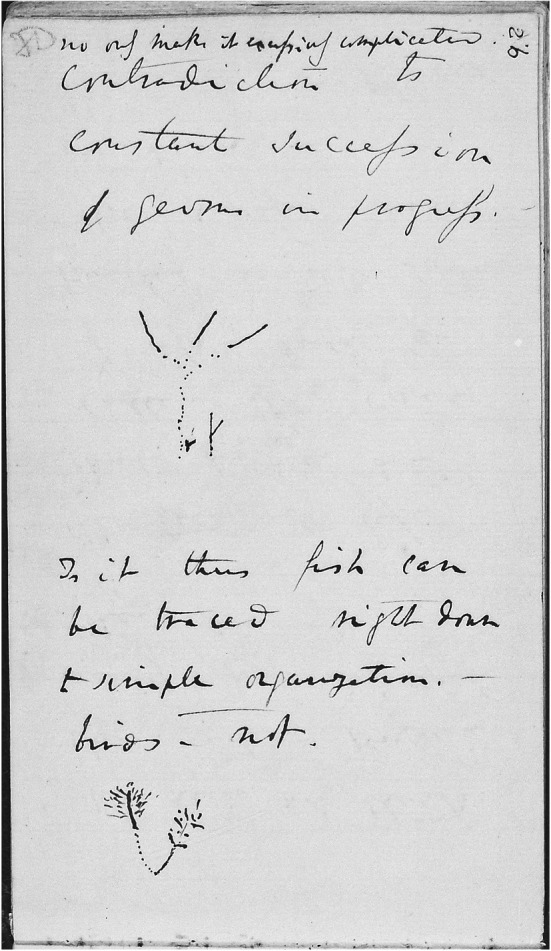
Page 26 of *Notebook B* (1837–1838). (Cambridge University Library MS DAR.121.26; reproduced by kind permission of the Syndics of Cambridge University Library.)

**Fig. 3:**
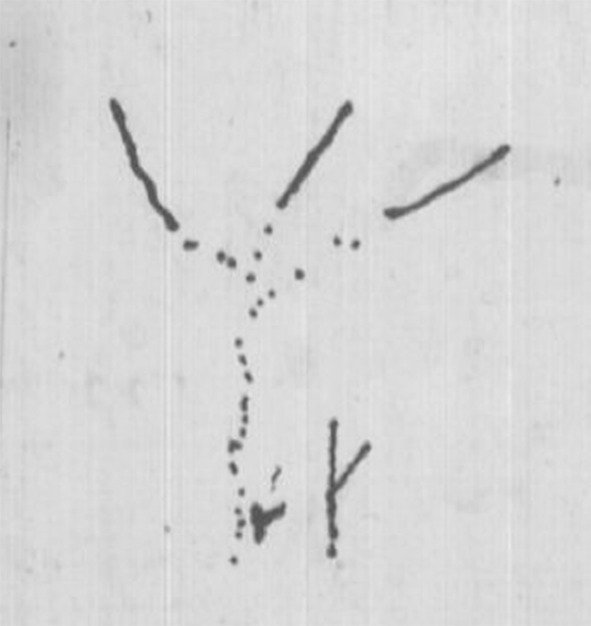
Detail of p. 26 of *Notebook B*

**Fig. 4:**
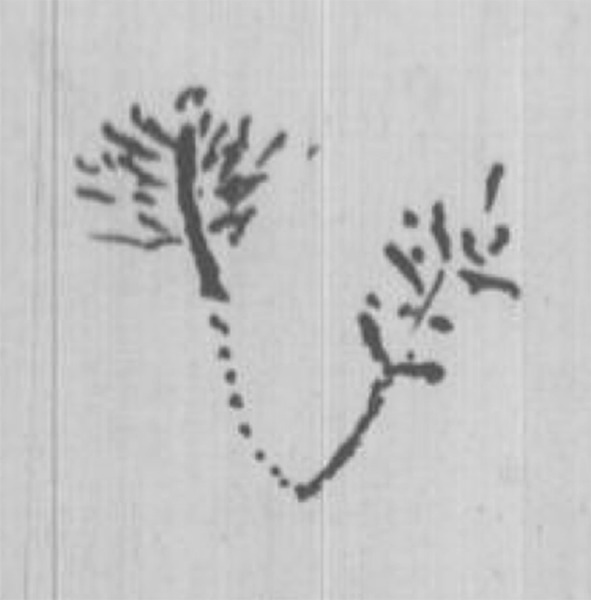
Detail of p. 26 of *Notebook B*

In July 1837, nine months after his return from the *Beagle* voyage, Darwin drew his first evolutionary sketches, found on page 26 of his Notebook B, which he had recently begun (Darwin 1837–1838). The graphic elements he used in composing all his sketches—points, lines, and spaces—are immediately identifiable (see Fig. 2 and, for more detail, Figs. 3 and 4).

**Fig. 5:**
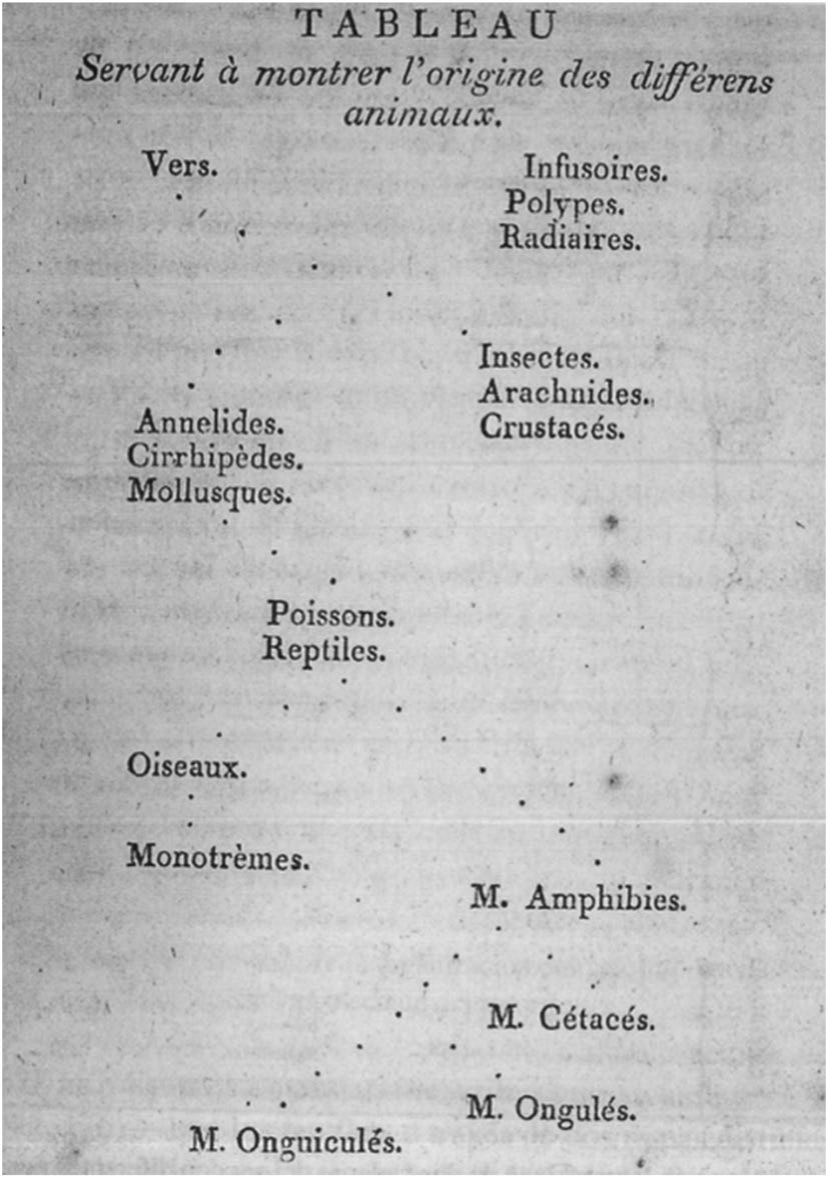
Lamarck’s “tree,” which purports to “show the origin of the different animals,” from *Philosophie Zoologique* (1809, p. 463)

These sketches were meant for personal use. Below we will ascertain whether they were phylogenetic, that is, depicted evolutionary descent. They were preceded by a limited visual tradition of trees of life made by prior authors meant for public use. These were, with one exception, not phylogenetic (Archibald 2009, 2014). The exception is the “Tableau servant à montrer l’origine des différents animaux” [“Diagram showing the origin of different animals”] that Lamarck included in *Philosophie Zoologique* (1809, p. 463; see Fig. 5). This figure is regarded as the first phylogenetic tree, although it is hard to discern a tree form in it (Archibald 2014, pp. 60–66; Tassy 2010, pp. 90–91). Lamarck obviously based this “tableau” on his own hypothesis of evolutionary development. Before comparing Darwin’s sketches to Lamarck’s “tree,” however, I will explore the meanings Darwin assigned to the points, lines, and spaces in his sketches by analyzing the text accompanying them.

On the cover of Notebook B, Darwin wrote: “Transmutation of Species.” This title heralded an important change in his interests and way of thinking. He had earlier believed that transmutation of species in nature—later to be called *evolution*—was a saltational process (Darwin 1859, pp. xiii–xiv). By 1837, he had abandoned this view, but it was only after he read Thomas Malthus’s *Essay on the Principle of Population* that he formulated the definite “theory by which to work” (Darwin 1859, pp. xiii–xiv). In the 25 pages in his notebook that precede the sketches, however, we see him trying to formulate a theory that can account for the transmutation and extinction of species. He is convinced that organisms are adapted to their environment: “We see the young of living beings, become permanently changed or subject to variety, according to circumstances … hence we see generation here seems a means to vary, or adaptation” (Darwin 1837–1838, p. 3). He also realizes that these adaptations are hereditary: “Beautiful law of intermarriages separating partaking of characters of both parents, and these infinite in number” (Darwin 1837–1838, p. 5). But, he wonders, does not the total number of species have to be approximately constant? “With this tendency to change (& to multiplication when isolated requires deaths of species to keep numbers of forms equable). But is there reason for supposing numbers to be equable” (Darwin 1837–1838, pp. 200–221). Imagining a tree—four pages later, he actually drew one—helps to give him the answer: “Organized beings represent a tree irregularly branched some branches far more branched — Hence Genera. —) As many terminal buds dying as new ones generated” (Darwin 1837–1838, p. 21).

Up to this point, Darwin had established how species are generated and also expressed the principle of equilibrium between the number of species generated and those that have become extinct, but he still has to clarify the cause of extinction. His explanation is based on monadism, the theory that simple living particles, or monads, are originating constantly in extremely large numbers in inanimate matter. These monads supposedly evolve in response to environmental conditions along branching paths into groups of related, more complex organisms (that is, species, which together form a genus). The monads have a definite lifespan; thus, when their life comes to an end, every species it has evolved into (and thus also the genus) must die too (Gruber 1974, pp. 136–137; Sloan 1986, p. 440; Kleiner 1981, p. 134). Hence the passage: “There is nothing stranger in death of species than individuals. If we suppose monad definite existence, as we may suppose in this case, their creation being dependent on definite laws, then those which have changed most owing to the accident of positions must in each state of existence have shortest life. Hence shortness of life of Mammalia” (Darwin 1837–1838, pp. 22–27).

Thus far Darwin’s words create the impression that he is recording a process in which thinking and writing, floating on a stream of consciousness, advance each other, but now he appears to need the additional means of providing a drawing to further the process. Before starting to use this new means of expressing his thoughts, however, he pauses for a moment to recapitulate the principal features of his developing theory that he wants to clarify by making sketches:

Would there not be a triple branching in the tree of life owing to three elements air, land & water … if each main stem of the tree is adapted for these three elements, there will be certainly points of affinity in each branch. A species as soon as once formed by separation or change in part of country repugnance to intermarriage increases it settles it. We need not think that fish & penguins really pass into each other. —The tree of life should perhaps be called the coral of life, base of branches dead; so that passages cannot be seen. this again offers contradiction to constant succession of germs in progress no [*sic*] only makes it excessively complicated. (Darwin 1837–1838, pp. 23–26)

In Fig. 3, we find a pilot study for Darwin’s later phylogenetic trees, which I will analyze in the following sections. Howard E. Gruber, referring to this sketch, suggested that Darwin’s encounters with archipelagoes near a continental land mass, notably the Galapagos, “helped him to begin to clarify the branching image.” The similarity and the simultaneous differences of fauna and flora on the continent and the islands, Gruber explained, “coupled with the small differences from island to island, can be translated quite directly into a taxonomic tree which looks a lot like a fragment of Darwin’s image” (1974, p. 135). An indirect indication supporting this contention can be found in the opening passage of Notebook B: “According to this view animals on separate islands ought to become different if kept long enough apart with slightly differing circumstances. — Now Galapagos Tortoises, Mocking birds, Falkland Fox, Chiloe fox, — Inglish [*sic*] and Irish Hare—.” (Darwin 1837–1838, p. 7). Darwin’s sketch, however, does not show different evolutionary developments on separate islands, but rather “branching … owing to three elements air, land & water,” caused by different environmental conditions in the various elements to which organisms are hypothesized to adapt, not one caused by different conditions on land in islands separated by sea (Gruber 1974, p. 135).

Darwin explains the difference between the continuous lines and the dotted ones appearing in both sketches as follows: “We may fancy according to shortness of life of species that in perfection, the bottom of branches deaden, so that in Mammalia, birds, it would only appear like circles, & insects amongst articulata. — but in lower classes perhaps a more linear arrangement” (Darwin 1837–1838, p. 27). He assumes here that species of higher organisms—mammals, birds, or certain insects (that is, the higher taxon of invertebrates with an exoskeleton and segmented bodies)—become extinct sooner than more primitive ones. The lines of development of lower classes therefore “show a more linear arrangement” in the sketch, whereas the organisms in the lines of higher species “appear like circles” (that is, points). Fossils are represented between alternating spaces, which indicate places where links are missing in the line of development that would otherwise be uninterrupted. Hence, Darwin switches, as quoted above, from a tree-like to a coral-like interpretation of both of his sketches: the base of coral is, after all, dead.

Figure 4, Darwin’s second-known pilot study for a phylogenetic node, shows the junction of the lines of adaptive development, departing from a single “simple organization”: the assumed common ancestor of fish and birds. That is why he writes: “Is it thus fish can be traced right down to simple organization. — birds — not?” (Darwin 1837–1838, p. 26). The line of development of fish—presumably these are more primitive organisms—is uninterrupted by extinct species, while on the left we see many extinct species in the line of development of birds. In the previous pages, Darwin had described the initial cause of the branching into the two separate elements (water and air, or rather land) and the subsequent stabilization of the separate development of both branches: “A species as soon as once formed by separation or change in part of country repugnance to intermarriage increases it settles it. We need not think that fish & penguins really pass into each other” (Darwin 1837–1838, p. 26). This means that the branchings higher up in this sketch are not caused by the different conditions in water and land. These branchings show Darwin’s belief in the separation caused by repugnance to intermarriage, which later became an important tenet in *Origin*.

There is, however, something strange here: many of the branchings are disconnected from their point of origin. Given what we know now about the spaces in the dotted lines, these discontinuities may be interpreted as missing links between extinct species. A strong argument for this interpretation is Darwin’s aforementioned view concerning the lifespan of species: the more developed an organism, the earlier he supposes it would become extinct. On the other hand, it may be argued that it is not very likely that as many missing links as drawn in the right-hand branch would occur while no species is missing in the lower part of the branch. An alternative explanation could be that this is an alternative way of drawing “separation” and “repugnance to intermarriage.” In the “An Endless Piece of Seaweed Dividing” (1843) section, we will encounter a more apparent case of this interpretation.

The form of the sketch in Fig. 4, as well as its location in the text—directly following the sentence “The tree of life should perhaps be called the coral of life”—indicate clearly that this sketch is meant to be a coral. Bredekamp asserts that it and all the sketches following it are corals: “Mit der Koralle besaß er ein Modell der Evolution” [“In the coral he found a model of evolution.”] (2006, pp. 20–21). Bredekamp perceives the shape of a coral in another sketch, even while acknowledging that Darwin there avoided drawing a coral’s defining elements: “Zwar hat Darwin in dieser Zeichnung mit den Punktlinien das entscheidende Element seines Korallenmodells vermieden, aber dennoch erinnert bei seinem Gebilde nichts an einem Baum” [“Even though Darwin avoided with the dotted lines in this drawing the defining element of his coral model, there is nothing in his structure which reminds us of a tree”] (2006, p. 38). I disagree with Bredekamp here. Darwin’s later sketches—except for one which Darwin likened to seaweed—were not all characterized by a concrete form, whether a coral or a tree. Instead, he abstracted his sketches from concrete natural forms and tried to utilize the explanatory power of their elements—that is to say, points, lines, and spaces—to clarify his ideas in the best way possible.

If we take a closer look at Lamarck’s above-mentioned evolutionary figure (Fig. 5), the first thing to observe is that it is constructed from the top downwards and shows the development of animal life from infusoria to primates. Second, its graphic structure and system are quite different from Darwin’s sketches. The points in Lamarck’s figure do not have a particular meaning; they are merely widely separated dots suggesting lines. These lines have the same function as the continuous lines in Darwin’s sketches, whereas the discontinuities in Darwin’s dotted lines, as previously noted, may be interpreted as missing links between extinct species. The bifurcations and the ever increasing spaces between Lamarck’s dotted lines seem to work in the same way as the branchings and the spaces in Darwin’s sketches—that is, they symbolize speciation.

Darwin’s figures differ from Lamarck’s dotted lines not only in form, but they also illustrate a different evolutionary hypothesis. Pascal Tassy has argued that Lamarck’s schema is the first “phylogenetic tree” published as such, because it aims to illustrate the relationship between taxa on the basis of their origin and history, as opposed to classification (2010, pp. 90–91). Archibald agrees, but argues that “Lamarck likely never accepted the idea that living forms have a common ancestor” and that for him, “what we call now evolution was an ongoing process of multiple spontaneous generations with bifurcations, yielding a tree that shows lines tracking through repeated divergences of one form to another as complexity increased” (2014, pp. 60–65).

It is clear that Darwin, in this phase of his thought process, ascribed the cause of evolution to a fundamentally different mechanism than Lamarck’s. What is witnessed here in Notebook B are Darwin’s first two attempts to utilize tree- or coral-like forms, to clarify speciation by adaptation and extinction as a consequence of monadism. Although historians know that Darwin was familiar with Lamarck’s ideas, he does not refer here nor anywhere else to Lamarck’s “tree,” and so we have no reason to conclude that he was influenced by it. In the next section, we will see Darwin trying to demonstrate through his drawings how old forms are pushed aside where speciation occurs.

## “I think **…**” (1837)

**Fig. 6:**
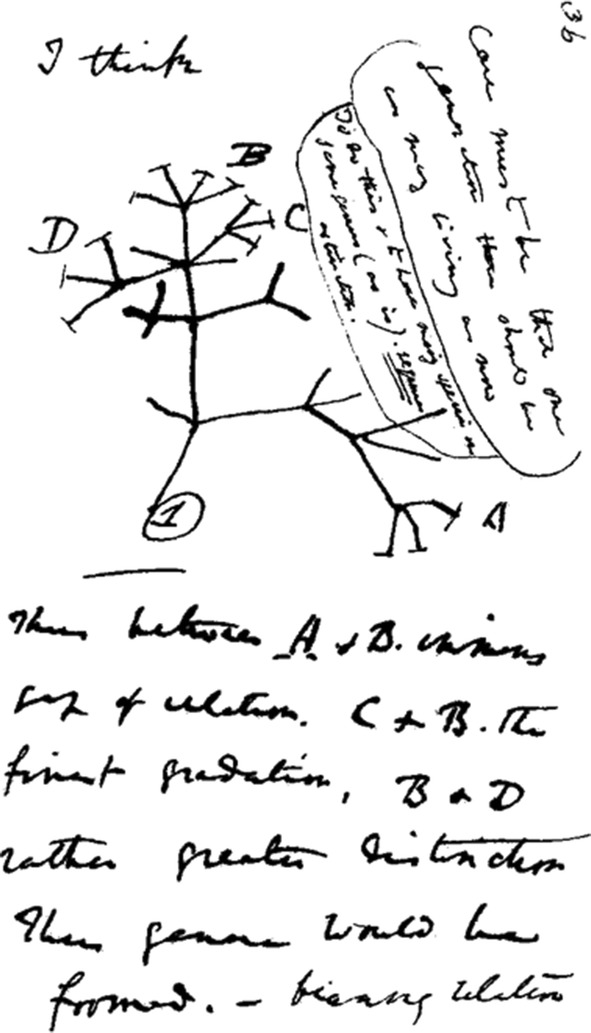
The “I think” sketch on page 36 of *Notebook B* (1837–1838). (Cambridge University Library MS.DAR.121.36; reproduced by kind permission of the Syndics of Cambridge University Library.)

In the summer of 1837, ten pages later than the earlier sketches in Notebook B, Darwin drew the “I think” sketch. His image was used to decorate all kinds of souvenirs sold to visitors to the great exhibition held in London during the 2009 Darwin Commemoration, and as a consequence, it became immensely popular among the general public. Looking at this small page (Fig. 6), together with the transcript of the text it carries, it is easy to assume that they track the formation of Darwin’s ideas (Gruber and Bödeker 2005, p. 160; Hodge 2003, p. 48). Darwin jumps from the midst of a thought—“I think”—directly to his sketch, which in turn generates new thoughts; these are laid down in two text balloons, the essence of which he transports in a concluding jump—*“thus”*—back into the textual flow of his stream of consciousness. It appears unlikely that the text balloons were added later on. Thus, I read the little page in the following order:

I think [sketch] [right-most text balloon:] Case must be that one generation then should be as many living now. [Text balloon right next to sketch:] To do this & to have many species in same genus (as is) requires extinction. [Text below sketch:] Thus between A & B immense gap of relation. C & B the finest gradation, B & D rather greater distinction. Thus genera would be formed. —bearing relation to ancient types. —with several extinct forms for if each species an ancient ① is capable of making 13 recent forms, twelve of the contemporarys must have left no offspring at all, so as to keep number of species constant. (Darwin 1837–1838, pp. 36–37)

We recall that the previous two sketches focused on principles that were related to specific conditions in nature (the three elements) or to organisms (fish or birds). The present sketch highlights an abstract level, and without referring to concrete matters, the discovery of two principles. The principle of dynamic balance between speciation and extinction appears in the text balloons, and the principle of evolutionary genealogical distance between descendants of a common ancestor in the evolutionary lineage (where Darwin would later find the underlying principle of divergence of characteristics) follows in the text immediately below the sketch.

It is noteworthy that, unlike his previous sketches, Darwin does not characterize this one as a living organism, such as a tree or a coral. Apparently, he is exclusively interested in the functionality of the sketch as such and of its elements. Such a functional approach verifies how Darwin uses the sketch, without the intermediation of a metaphor, so as to demonstrate to himself the principle of the dynamic balance between speciation and extinction.

The symbol ① in the figure is also mentioned in the quote above: “an ancient ①.” It represents an “ancient,” that is, ancestral organism whose offspring remains stable from one generation to the next, until the point in time (in fact, a phylogenetic node) when, supposedly as a consequence of the previously formulated principle of speciation by adaptation, its progeny starts to fan out in three directions. The offspring to the left become extinct. This is indicated by the absence of a final crossbar. To the right, we see a phylogenetic node which produces two extinct species and one continuous line of development. This line leads to a second node which produces two extinct species and a continuous line, which culminates in three species, whose terminal crossbars indicate that they will be able to produce extant offspring. Higher up, we see a similar development leading to more numerous progeny of ultimately extinct or surviving species. Tracing the development in Darwin’s assembly of line segments in this way, we may establish that it, unlike the previous sketches, enables him to show himself how extinction makes room for the selective survival of species. He achieves this by introducing the new graphic element previously mentioned of the final crossbar, which indicates a surviving species. Thanks to this device, he needs only to place crossbars at around half of the extremities of the sketch to show how the dynamic balance between speciation and extinction works.

But isn’t Darwin’s sketch a drawn version of the fallacy of begging the question? Doesn’t Darwin get out of his sketch what he first put in? After all, if one gives half of the extremities a crossbar, then inevitably the other half does not have one. At first sight, he seemed to say this himself in the above cited quote: “If each species an ancient ① is capable of making 13 recent forms, twelve of the contemporarys must have left no offspring at all, so as to keep number of species constant.” This is reminiscent of a stock management system which ensures that as many items enter a stock as those leaving it. But I still think that the sketch meant more to him than that. The jump from “I think” to the sketch shows that he experienced “the extraordinary difficulty,” quoted above, “which naturalists have … in describing, without the aid of a diagram, the various affinities which they perceive between the many living and extinct members of the same great natural class” (Darwin 1859, p. 431). Moreover, if it is the case that he made the sketch first and the text balloons after—and I think this sequence is obvious—then this demonstrates his need to gather his thoughts by drawing the sketch. According to his ideas on the balance between speciation and extinction, the latter is simply a process by which the generation of species is fatally crowded out by more successful ones due to a lack of space. In Fig. 10, Darwin draws in a more visually convincing manner how lack of space in a sketch can illustrate extinction due to lack of space in natural conditions. This way of drawing would eventually find its way to the diagram in *Origin*.

As previously stated, Darwin also utilized the sketch to demonstrate to himself the second principle, that of an evolutionary genealogical distance between descendants of a common ancestor. To do this, he needed only to place the letter A near the crossbar at one extremity of the right-hand branch and to distribute the letters B, C, and D to three surviving species at the crossbars at the other branch in the upper part of the sketch.

Darwin’s conclusion about the result seems strange in light of his previous observations that B and C are most closely related, that B and D are less closely related, and that the gap of relationship between A and B is immense. The latter conclusion is obvious, but it is difficult to understand why B, C, and D should not be equidistant, for they are all separated from one another by the same number of nodes. Archibald suggests that the distinction made by Darwin arises from the convention at the time of showing closeness of relationship by the relative position on the tree and not just by relative branching (2014, p. 83). A problem with this explanation is the relative position of A on the tree in relation to its ancestor ①. Only one less node separates it from the common ancestor than separates B, C, and D, but it is positioned equally high in the figure. This ambiguity remains unresolved.

**Fig. 7:**
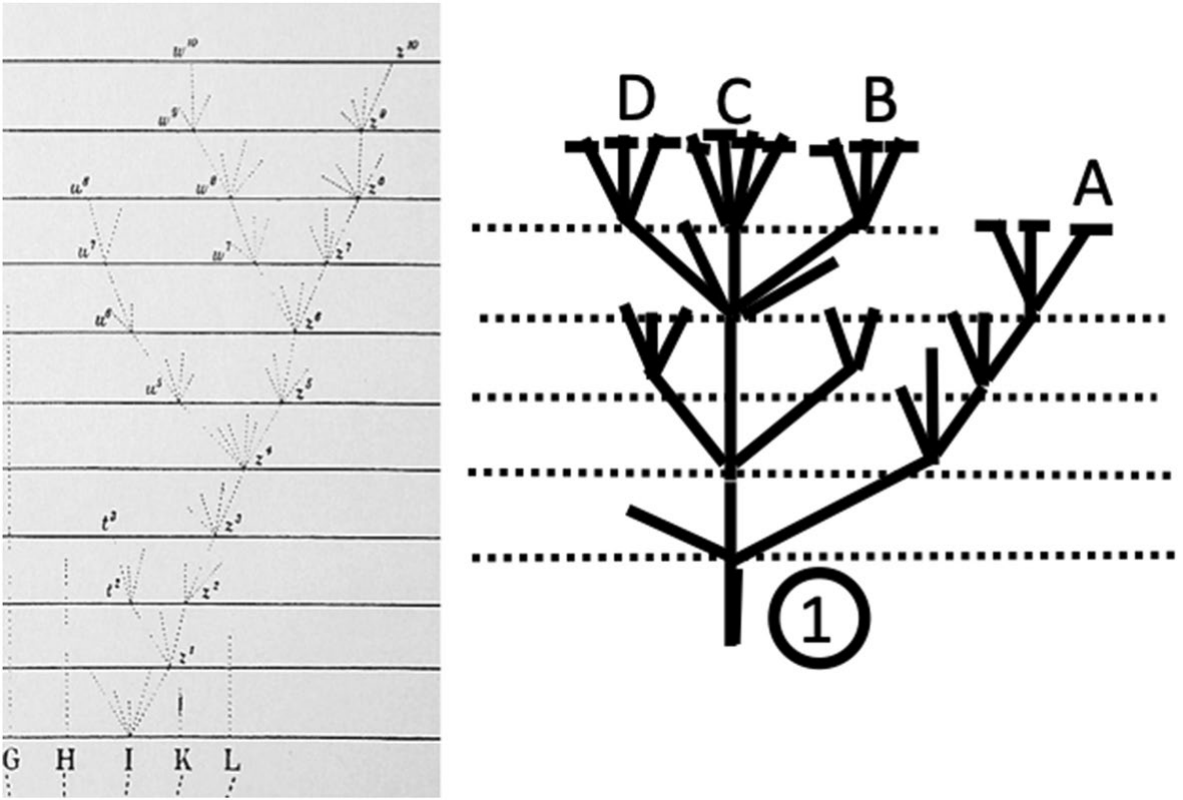
The “I think” sketch, bent upward and compared to a fragment of the diagram in *Origin*

Archibald calls this sketch a “branching stick figure” (2014, p. 82). With regard to its form, this characterization is more apt than that of a tree or coral, but in naming it thus, he overlooks the centrifugal notion of time of the sketch. This notion follows from the fact that time elapses outward along its branches, which grow in opposite directions, thus seemingly representing the ever-decreasing evolutionary affinity between the species on the branches. It is not clear whether Darwin was aware of the temporal implications. This aspect of the sketch could have been unintentional; neither the text in the *Notebook* nor any other source provides any information on this. However, as we will see in “Lines and Points in Geological Space-Time (1857)” section, in 1857 Darwin consciously and deliberately drew a centrifugal 360-degree space-time sketch in order to create room for similar diametrical evolutionary developments and extinctions. To incorporate that sketch, and what it taught him about the above-mentioned aspects of evolution and about fossilization, in the diagram in *Origin*, he had to bend it upward by transferring the lapse of time to the vertical axis of the diagram. Reworking the “I think” sketch in the same way, the result (Fig. 7) appears to show a structure that strongly resembles the pattern of the diagram in the *Origin*. It appears abundantly clear that here Darwin demonstrates two of the principles of his theory, simply by using lines and letters, in almost the same way he will do in his diagram in *Origin* twenty-two years later.

The result also resembles the phylogenetic trees that later became indispensable to evolutionary biologists. Of course, bending and deforming the “I think” sketch does not make it a phylogenetic tree. It is, in fact, the other way around: in its original form, the sketch is the prototype—an early sample of a product meant to test a concept or process or to act as a thing to be replicated or learned from (Blackwell and Manar 2015)—of the phylogenetic tree.

## “An Endless Piece of Seaweed Dividing” (1843)

It was not until 1839, two years after drawing the “I think” sketch, that Darwin finally came to the conclusion that natural selection had to be the driving mechanism of transmutation, thus completing his “theory by which to work.” This event as such falls outside the scope of this article, given that, at that moment, as far as we know, no sketch was involved.

**Fig. 8:**
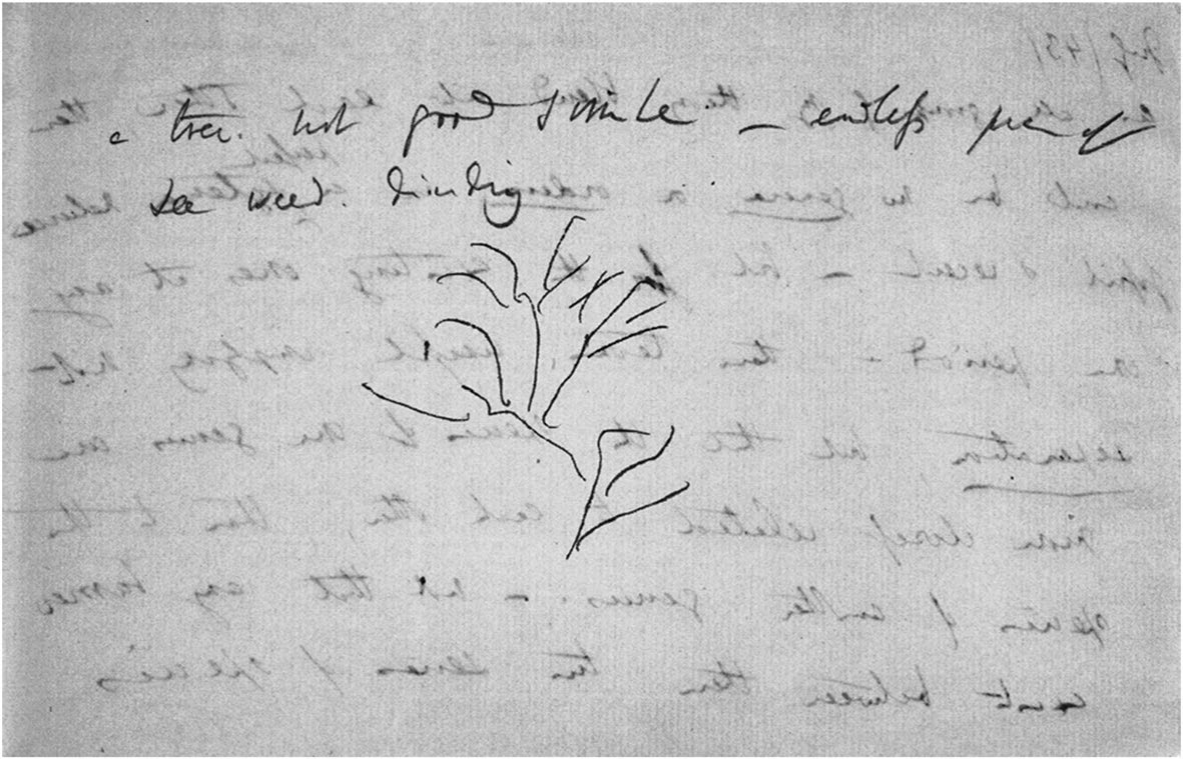
Darwin’s sketch of seaweed drawn on a loose sheet of paper (1843). (Cambridge University Library MS.DAR.205.5.90v; reproduced by kind permission of the Syndics of Cambridge University Library)

For a long time, it has been assumed that Darwin, having formulated the principle of natural selection, then focused his energy on subjects not directly related to his theory: for instance, between 1846 and 1854, on his research project on barnacles. However, recent scholarship on Darwin’s barnacle study discusses the role this work played in facilitating his understanding of the vast wealth of variations on which selection works, which supported his theory (Love 2002, pp. 269–281; Stott 2003; Richmond 2007). Anyhow, a drawing related to the marine environment of barnacles, made even earlier, in 1843, attests to the fact that his thinking about evolution did not stand still. What he drew then does not grow on land but in the sea. On a piece of gray paper, he doodled sixteen lines; above the resulting sketch, he wrote: “a tree not good simile—endless piece of seaweed dividing” (Fig. 8).

On the reverse side, he described the point he obviously wished to clarify: “As all groups by my theory blend into each other, there could be no genera or orders «in same sense that no part of a tree can be said to be distinct» in a «perfect» systema naturae fossil & recent—but for the existing ones at any period—these terms useful, implying not separation, but that the species of one genus are more closely related to each other, than to the species of other genus.—not that any barrier exists between these two series of species / over”.^4^ Archibald states, “One cannot know with certainty Darwin’s thoughts here, but it would seem that he still struggled with the notion of all life through time as continuous so that no metaphor—trees, corals, or seaweeds—captured his vision” (2014, p. 85). In contrast, I believe that it is possible to move much closer to Darwin’s thoughts, and that he meant that in this case the simile of seaweed was adequate.

The sketch consists of only one graphic element: sixteen lines that are not connected to each other (except right at the bottom and once high in the middle) and crossing one another only once. The sketch resembles, in a certain sense, the upper parts of Fig. 4, where many of the branchings are disconnected from their branch of origin. There I surmised that these discontinuities represent extinct species; here, however, it seems more as if all the side branches are disconnected from a main branch. Finally, the lines are not straight but curved.

The caption of the sketch, “a tree not good simile—endless piece of seaweed dividing,” indicates that Darwin needs an alternative way of sketching an aspect of evolutionary development, which is characterized by two key concepts: “endless” and “dividing.” *Macrocystis pyrifera*, or kelp, the largest of all algae, meets these concepts best. Darwin knew this species of perennial seaweed from his own experience. He had become acquainted with it in June 1834, when the *Beagle* was cruising along the western shore of Tierra del Fuego in Chile. On 1June he noted that he had observed “kelp, or Macrocystis pyrifera. This plant grows on every rock from low-water mark to a great depth, both on the outer coast and within the channels.” He mentioned the fact that it could reach a length of almost 110 meters: “I do not suppose the stem of any other plant attains so great a length as three hundred and sixty feet, as stated by Captain Cook” (Darwin 1845, p. 239). Admittedly, this is not “endless,” but it is evident that in the context of this sketch, the word “endless” is not meant to be taken literally. The interpretation of the word “dividing” depends on one’s view of the disconnections between the branches. Two views are possible here: one is that the piece of seaweed was so hastily drawn (as might possibly be inferred from the jerky curves of the lines) that being attached or not is a matter of chance, not of choice, while the other is that Darwin deliberately drew branches that are disconnected from their stem of origin.

If the latter is the case, the disconnections can be traced back to the fact that kelp, supposedly the chosen simile, is a perennial seaweed in which the secondary and tertiary branches divide annually from the original stem. This suggests that, unlike the discontinuities in the coral similes of Figs. 3 and 4, the discontinuities in the seaweed sketch do not represent extinct species, but another phenomenon.

This suggestion is confirmed by the fact that on the reverse side of the paper, as noted above, Darwin writes about something completely different: the fact that, according to his theory, “all groups blend into each other,” and the problem that it is therefore impossible in principle to distinguish between genera. “There could be no genera or orders «in same sense that no part of a tree can be said to be distinct.»” He refers here to the imperceptibly small steps of evolution. Later on, in *Origin*, he would refer six times to the canon of “natura non facit saltum” (Darwin 1859, p. 471). The small differences mentioned now, in 1843, on this small piece of paper make it impossible to distinguish genera “but for the existing ones at any period.”^5^ But if a separation between genera is lacking, how can one imagine that speciation is caused by crossing species *within* a genus? Darwin’s answer on the reverse side is “that the species of one genus are more closely related to each other, than to the species of other genus.—not that any barrier exists between these two series of species / over” Thus, he points ahead to his doctrine of the aversion of organisms in nature to interbreeding (Hodge 2003). The sketch helps him to show this. The shedding of a secondary branch represents a genus “dividing,” while the original one in the stem remains the same, and, as Darwin will later put it in *Origin*, “may for a long period continue transmitting unaltered descendants” (Darwin 1859, p. 121). Thus, these “endless” wavy lines are a premonition of the ascending lines in the diagram, which show this aspect.

In the other interpretation, where the “piece of seaweed” was so hastily drawn that being attached or not is a matter of chance, not of choice, it is sensible to suppose that Darwin intended to draw nodes of separation, but failed to do this due to insufficient pen control. In this case, “dividing” means that after the imperfectly drawn nodes where two genera separate, their branches continue on their own, “transmitting unaltered descendants.” The fact that they are supposedly doing this “endlessly” can have been the only reason why Darwin would have preferred the simile of an “endless piece of seaweed dividing” over that of a tree in this case.

In view of the latter, it seems obvious to suppose that Darwin drew deliberately disconnected branches. It should be noted, however, that the result of this way of “dividing” would have to be that the genus of the stem itself continues “unaltered,” while that of the separating secondary branch becomes a different one. This modus of “dividing” did not find its way to *Origin*, neither to the diagram nor to the text. This does not imply that Darwin could not have been playing with the idea of this modus of speciation; it simply means that, in contrast to the idea of lines “endlessly transmitting unaltered descendants,” it did not survive in his thinking. However, the question of what modus of “dividing” Darwin had in mind when drawing the seaweed remains unanswered.

This is the first and only appearance in the series of a metaphorical seaweed. The seaweed sketch, then, as regards form and content, is unique in the series of sketches and helped Darwin, when ordering his thoughts on speciation, to arrive at one clearly identifiable element of his diagram.

## A Universal Law Is Not Found (1848)

As mentioned in the previous section, in 1846 Darwin began work on a huge research project on barnacles, that would take eight years to complete. In a letter to Syms Covington, his former servant, he defined barnacles as “conical little shells, with a sort of four-valved lid on the top. There are others with long flexible footstalk, fixed to floating objects, and sometimes cast on shore”^6^ (Browne 1995, p. 486). Darwin remained occupied with this project until the early 1850s; it resulted in four big monographs on the taxonomy of living and fossil barnacles (Browne 1995, p. xiv; Love 2002, pp. 269–281; Stott 2003; Richmond 2007).

The barnacle family is a vast one, comprising many genera, each of which consists of countless species. Its nomenclature was a confusing tangle, and Darwin often found this research a torment. In 1849, he wrote to the botanist Joseph Dalton Hooker, his closest friend: “I have of late been at work at mere species describing, which is much more difficult than I expected…. What miserable work, again, it is searching for priority of names; I have just finished two species which possess seven generic & 24 specific names.”^7^ The interesting point here is that in this “miserable work,” Darwin seems to have been confronted by the diffuse borders in nature between and within species and genera in this marine family. But because he had embraced the principle of phylogeny in 1837, with the considerable help of the “I think” sketch, classification for him meant tracing evolutionary lineages in phylogenetic relations. Darwin stressed this point in an 1843 letter to the naturalist Robert Waterhouse:

According to my opinion, (which I give every one leave to hoot at, like I should have, six years since, hooted at them, for holding like views) classification consists in grouping beings according to their actual relationship, ie their consanguinity, or descent from common stocks—In this view all relations of analogy &c &c &, consist of those resemblances between two forms, which they do not owe to having inherited it, from a common stock.—To me, of course, the difficulty of ascertaining true relationship ie a natural classification remains just the same, though I know what I am looking for.^8^

**Fig. 9:**
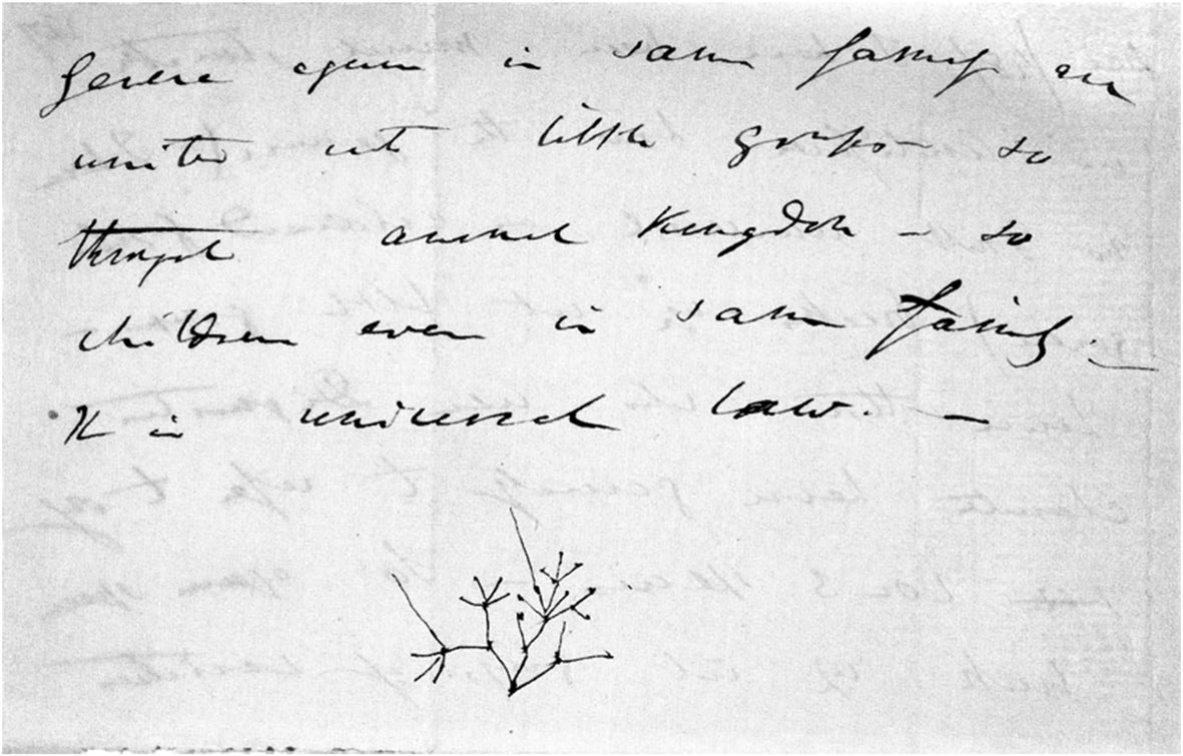
Sketch on a loose sheet accompanied by observations about how genera break up into groups (1848) (Cambridge University Library MS.DAR.205.5.127v; reproduced by kind permission of the Syndics of Cambridge University Library)

In 1848, two years after he started working on the barnacle project, he made a tiny sketch (Fig. 9).

The text related to the sketch reads:

“Dec. /48/. I have been much struck in Anatifera how the genus, (& I have no doubt universal, as evidenced by sub-genera) breaks into little groups—hence those who use Di[a]gnostic character have generally to refer to only 1 or 2 or 3 species—So again species break up into groups of varieties [verso side] Genera again in same family are united into little groups—so throughout animal Kingdom—so children even in same Family—It is universal law”.^9^

The “Anatifera” mentioned in the text are one of the species of barnacles Darwin was classifying: *Lepas anatifera* in the family Lepadidae. Important in the text is Darwin’s observation that the many differences within the abundance of varieties, species, subgenera, and genera must be caused by a universal law. It seems a reasonable assumption that at this moment, during his barnacle project, he resorted to sketching in order to generate ideas about this law. In 1843, Darwin used a sketch to explain the origination of species where the undefinable boundaries between genera seemed to make speciation impossible. Now in 1848, while classifying barnacles, Darwin pondered another aspect of speciation: namely, the suspected universal law that was supposed to cause the successive fragmentation and radiation of families into genera, into species, and into varieties (Fig. 9).

This sketch resembles the “I think” sketch, except that Darwin indicates the nodes by points, which are also used at the termini of some branches instead of crossbars. Another difference is the extension of two branches well beyond the other ones. These first appear to be longer lines of barnacle species that, like the stable species we met in the previous section, which as Darwin will later put it in *Origin*, “may for a long period continue transmitting unaltered descendants” (Darwin 1859, p. 121). In contrast, the four-tiered structure of family, genus, species, and variety, mentioned in the text, is clearly evident in the ascending middle part of the sketch. The polytomies in this part—one showing five branches and one showing three—also emphasize the fragmentation, mentioned in the text, of small groups which in turn break into smaller groups, but it is not clear with which specific groups Darwin is dealing.

But no matter how we look at it, the sketch did not help Darwin to uncover the universal law to which he refers in his text. Indeed, this sketch is the only one in the series that did not help Darwin’s thinking process. Many years later, in Chapter 5 of *Origin,* it is apparent that in the meantime he had discovered not one law, but several causes of variation, labeled “Laws of Variation” in the chapter’s title.

## “The Embryo is the Animal in Its Less Modified State” (1852–1855)

**Fig. 10:**
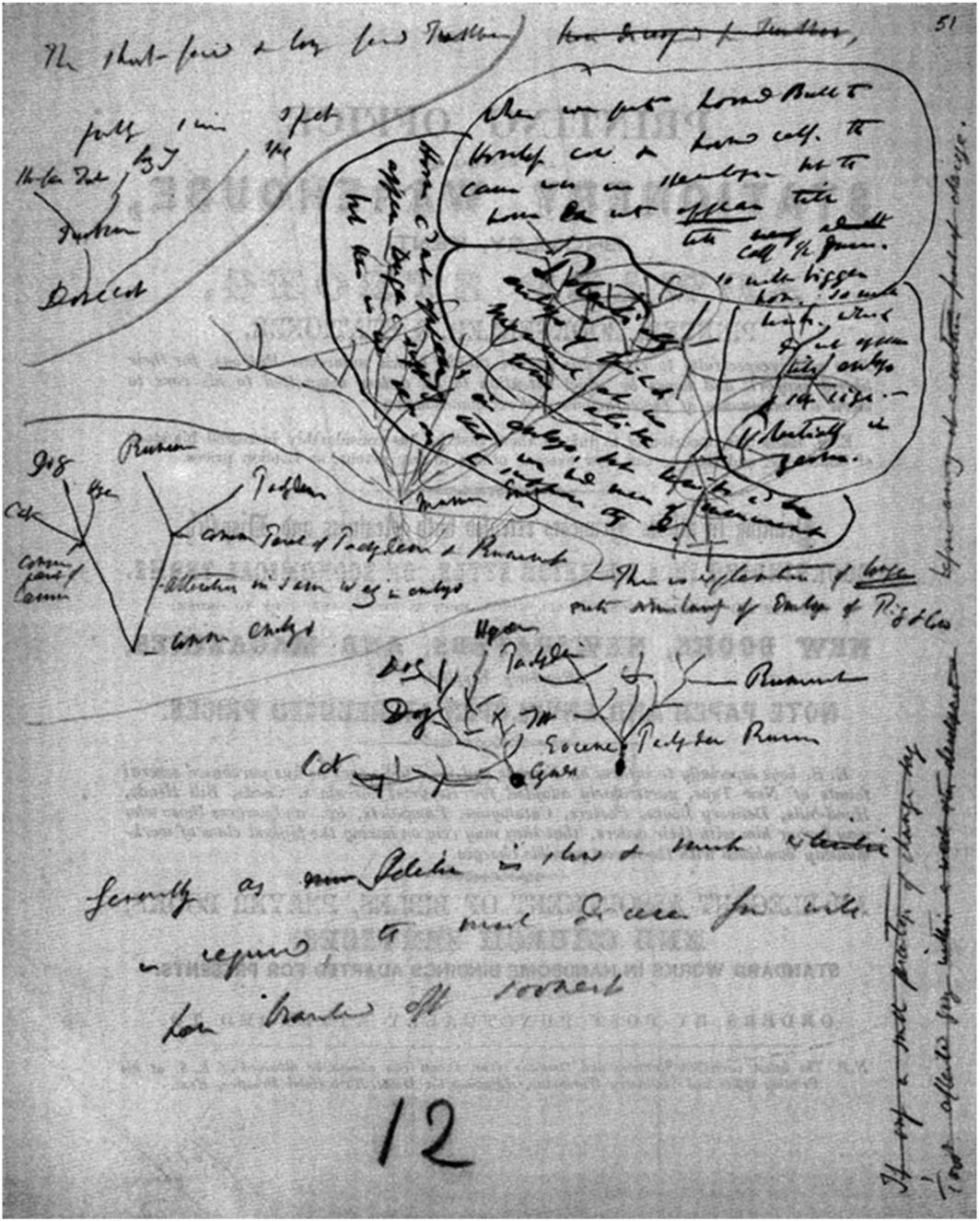
Five sketches, depicting embryological genealogy on the blank side of an advertising leaflet. (1852–1855) (Cambridge University Library MS.DAR.205.65lr; reproduced by kind permission of the Syndics of Cambridge University Library.)

**Fig. 11:**
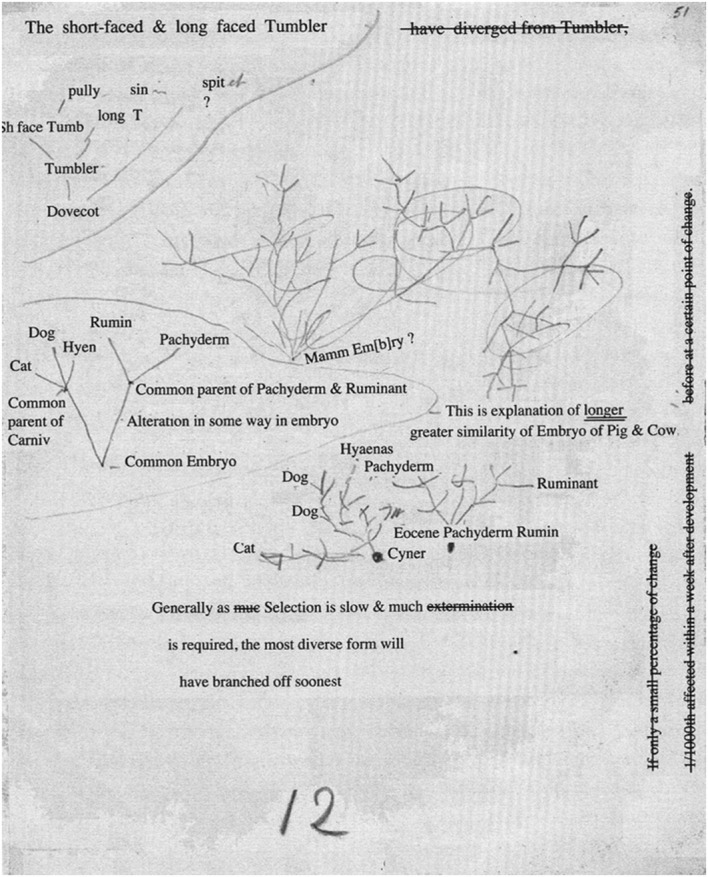
Transcription of the text in Fig. 10. (Archibald 2014, p. 94; reproduced by kind permission of J. David Archibald.)

Sometime between 1852 and 1855, Darwin used the back of an advertising leaflet for the Edward Strong Printing Office and Stationery Warehouse to record, in a tangle of lines and illegible handwriting, his thoughts about the early stages of the evolution of mammals (Figs. 10 and 11). In the upper right corner, there are three text balloons drawn across a treelike structure. Near its stem we find the words “Mamm Em[b]ry.*”* Surrounding it, four smaller, similar structures appear. Near the origin of one of these, there is the text “common embryo.”

The structures I have analyzed in the previous sections all appeared to be phylogenetic trees—that is, diagrams representing the evolutionary lineage of species or higher taxa. In this case, by contrast, these are structures wherein the phylogeny—or the lineage of species—is represented by using the classification of embryos. My supposition is supported by what Darwin himself says on this subject in *Origin*:

Descent being on my view the hidden bond of connexion which naturalists have been seeking under the term of the natural system. On this view we can understand how it is that, in the eyes of most naturalists, the structure of the embryo is even more important for classification than that of the adult. For the embryo is the animal in its less modified state; and in so far it reveals the structure of its progenitor. In two groups of animal, however much they may at present differ from each other in structure and habits, if they pass through the same or similar embryonic stages, we may feel assured that they have both descended from the same or nearly similar parents, and are therefore in that degree closely related. Thus, community in embryonic structure reveals community of descent. (Darwin 1859, p. 449)

It is tempting to think that Darwin refers here to recapitulation, the hypothesis that embryonic development is an accelerated repetition of the species’ evolution as a whole, later summarized by Ernst Haeckel’s maxim “Die Ontogenese rekapituliert die Phylogenese” [“ontogeny recapitulates phylogeny”]. But this is not the case. In the footsteps of Karl Ernst von Baer, Darwin rejected the concept of linear recapitulation.^10^

If Darwin could corroborate his hypothesis, then embryology would support his attempt to achieve “consilience of inductions,” the nineteenth-century methodological principle that converging evidence from independent, unrelated sources is the basis of strong conclusions. That is why, in *Origin*, he composed a nine-page argument to support his idea about similarities in embryonic structures of different species as indicators of common evolutionary lineage.

**Fig. 12:**
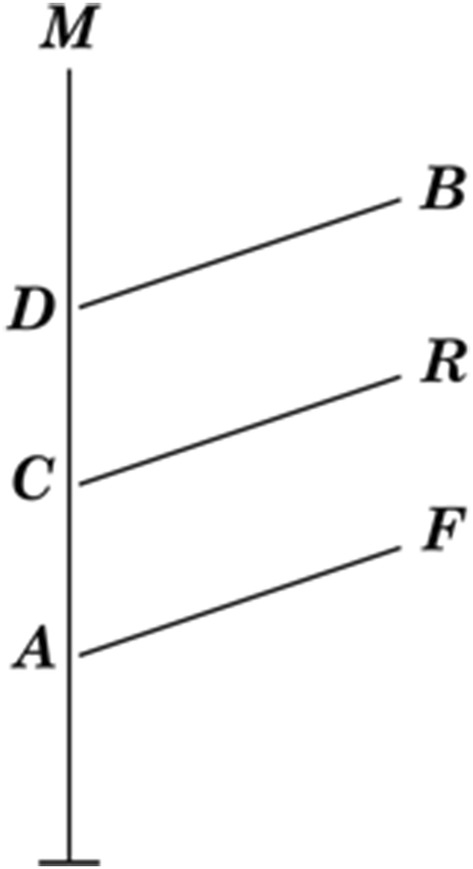
Robert Chambers’s diagram from *Vestiges of the Natural History* (1844), illustrating how embryological changes can be interpreted as evolutionary development. It is based on William Carpenter’s almost-similar diagram from *Principles of General and Comparative Physiology* (1841), which shows differences in timing of embryological development (Secord 2000, p. 76; Archibald 2014, p. 66; Pietsch 2012, p. 76)

Prior to Darwin’s sketch-supported musings on embryology, Robert Chambers anonymously published his *Vestiges of the Natural History* in 1844. In this book, he promoted a miscellaneous theory of general progression in which he combined, among other items, the idea of stellar evolution with an idiosyncratic theory of transmutation of species. This “hypothesis of the development of the vegetable and animal kingdoms” can be summarized in the author’s own words: “God created animated beings …,” and also a process in which “the simplest and most primitive type … gave birth to the type next above it, that this again produced the next higher, and so on to the very highest …” ([Chambers] 1844, p. 222). The book was well written, and it created, as James Secord wrote, a “Victorian Sensation” among the public, but was regarded as unscientific by biologists, including Darwin (Secord 2000, pp. 433–532; Archibald 2014, p. 68; Schwartz 1999, p. 132). Chambers demonstrated his theory through a diagram (Fig. 12). The diagram represents a distorted and misunderstood version of “von Baer’s Law” of embryological development. This is what the diagram shows, in Chambers’ own words:

The foetus of all the four classes may be supposed to advance in an identical condition to the point A. The fish there diverges and passes along a line apart, and peculiar to itself, to its mature state at F. The reptile, bird, and mammal, go on together to C, where the reptile diverges in like manner, and advances by itself to R. The bird diverges at D, and goes on to B. The mammal then goes forward in a straight line to the highest point of organization at M. ([Chambers] 1844, p. 212)

Secord analyzed Darwin’s reaction to the *Vestiges* and concluded that he profoundly disagreed. Darwin believed that Chambers promoted a simplified version of evolution, in which new forms immediately originated from old ones without intermediate stages and “the idea of a Fish passing into a Reptile [is] monstrous” (Secord 2000, pp. 431–433). This interpretation rules out the possibility that Darwin had been inspired or influenced in any way by Chambers’s theory. He had no need to adopt Chambers’s visual strategy, either, because as we have seen, he had already developed his treelike figures consisting of branches and branchings.

By contrast, in *Origin* Darwin sought to demonstrate that embryos of related species share common features, and that differences between their mature organisms are caused by later modifications in the individuals (Darwin 1859, p. 444). He opened his argument by showing that it is very hard to predict whether roughly similar juvenile organisms will develop differently, illustrating this with an example taken from the domestic environment. Just as cattle breeders or stud farmers cannot predict the characteristics of calves or foals until a certain time has expired after their birth, so we ourselves—he means Victorian gentlemen—can accurately predict the ultimate height of our children only during their adolescence (Darwin 1859, p. 443). Nevertheless, Darwin wrote, characteristics developing later on in youth have in many cases doubtlessly already been caused before or during the formation of the embryo: “An effect thus caused at a very early period, even before the formation of the embryo, may appear late in life … as when the horns of cross-bred cattle have been affected by the shape of the horns of either parent” (Darwin 1859, p. 443).

With this in mind, the uppermost text balloon of Fig. 10 reads: “When we put horned Bulle to Hornless cow & horned calf. the cause was in embryo [?]—but the horns do not appear till nearly adult calf 1/2 is grown. so with bigger horn, so with link, which do not appear till embryo of same size.—Potentially in germ.” And in the lower left balloon: “Horns cd not appear & in embryo, but limbs cd be appear longer, supposing that we had means of measurement, but there is no reason to suppose they do”.^11^ It seems evident that these notes prefigure his argument in *Origin*, and that appears to hold true for all the text and most of the sketches in Figs. 10 and 11. A strong example is the small tree in the upper left, aptly rooted in a “Dovecot.” This idiosyncratic rooting is possibly derived from Darwin’s reading of Edmund Saul Dixon’s 1851 book “The Dovecote and the Aviary.” Inspired by Chambers’s theory, Dixon argued that the development of the modern types of pigeons could only have occurred if completely new species had been found hatching in dovecotes (Dixon 1851, p. 76). Darwin, who in *Origin* demonstrated that he was extremely interested in pigeon breeding, knew better on the basis of his own experience and his theory, and so in the margin of the article he wrote curtly “no” (Di Gregorio 1990, p. 200; Secord 2000, p. 433).^12^ The “Dovecot”-tree sketch was apparently meant to demonstrate that he was right. Above it he wrote: “The short-faced & longfaced Tumbler have diverged from tumbler.” Evidently the structure and the words refer to dove races. In the passages in *Origin* devoted to embryology, he makes the following observations on pigeon breeding: “As the evidence appears to me conclusive, that the several domestic breeds of Pigeon have descended from one wild species, I compared young pigeons of various breeds, within twelve hours after being hatched” (Darwin 1859, p. 445). Measuring and weighing juvenile doves of distinct races, he appears to have been unable to note any discrepancy, while there is a world of difference between mature doves. However, “there was one remarkable exception to this rule, for the young of the short-faced tumbler differed from the young of the wild rock-pigeon and of the other breeds, in all its proportions, almost exactly as much as in the adult state” (1859, p. 445). The conclusion is that sometime between 1852 and 1855, Darwin hatched elements of the “long argument” later to appear in *Origin,* with the help of these small, phylogenetic, treelike structures.

The lowermost sketch shows something similar. Near its origin, Darwin wrote “Cyner.” Obviously, he meant an ancestor race of wild dogs, for in *Origin* he wrote: “The greyhound and bulldog, though appearing so different, are really varieties most closely allied, and have probably descended from the same wild stock” (Darwin 1859, pp. 444–445), and here we meet greyhound and bulldog in the sketch, somewhat surprisingly, in the company of cats.

The group gathered in the middle left sketch is even more heterogeneous. Here the supposed common lineage deduced from embryology appears: cats, dogs, hyenas, ruminants, and pachyderms (the latter being thick-skinned animals, a now obsolete order of mammals similar to elephants, rhinoceroses, and hippopotamuses). To enable this minor tour de force of evolution, Darwin has to employ a kind of subterfuge: the assumption that, in the common evolutionary branch leading to pachyderms and ruminants, an “alteration in some way in embryo” must have occurred.

The lower right structure depicts the supposed common ancestry of pachyderms and ruminants. Here, Darwin supposed the existence, in the Eocene, of a thick-skinned ruminant that must have been the common ancestor of both ruminants and pachyderms. No traces of the possible contribution of these last two sketches to Darwin’s reflections are detected in *Origin.* This is not at all surprising, for it is hard to substantiate that the kinship he erroneously depicted in the sketches could have been inferred from embryonic similarities of thick-skinned juveniles and calves.

The other three structures Darwin sketched on the blank side of this advertisement, however, apparently helped him in the process of iteratively thinking, drawing, and writing, a phenomenon we have observed in the previous sections. Their contribution to the process must have been effective, because the results can be found in passages in *Origin.* That the three structures could help crystallize Darwin’s ideas was apparently due to the flexible way he phylogenetically represented evolutionary development in nature, for in this case his sketches seem to be able to depict the phylogeny of embryos and juvenile mammals.

## “The Parent of Marsupials and Placentals” (1857)

**Fig. 13:**
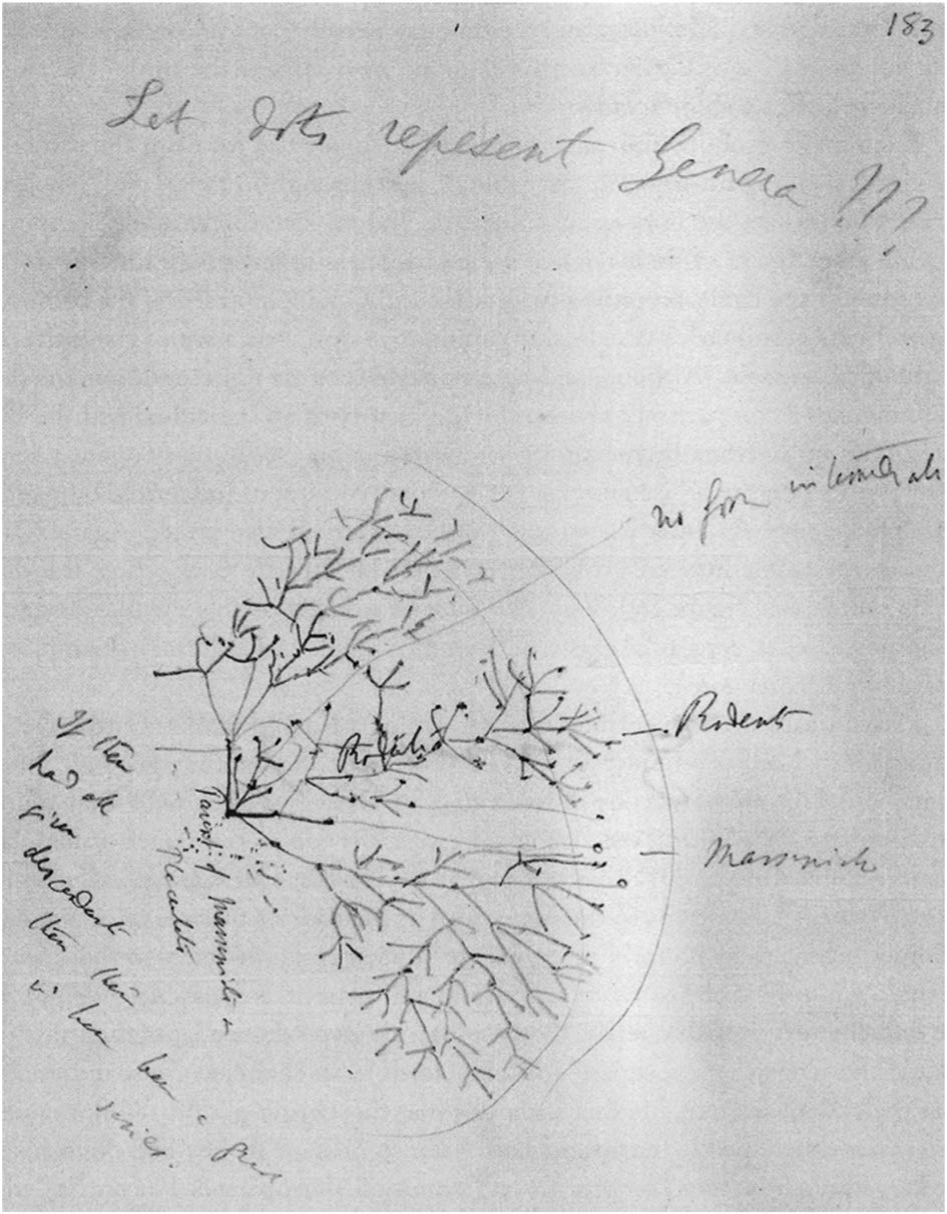
Sketch on a loose sheet depicting the phylogeny of mammals and marsupials and the cause of extinction (dated circa 1857). (Cambridge University Library MS.DAR.205.5.183r; reproduced by kind permission of the Syndics of Cambridge University Library.)

Over the years, Darwin’s evolutionary sketches became more complex, as can be seen in a sketch he drew in 1857 or 1858 (Fig. 13). As Voss correctly remarked, Darwin put every element of his drawings to the test with increasing frequency, by raising questions in countless letters to correspondents all over the world, by studying an equally countless number of books, by running experiments in his garden, and by making statistical analyses (2010, p. 108). All of these elements appear in the sketch he drew under the heading “Let dots repesent [*sic*] Genera???”^13^

To the right of the drawing, Darwin wrote “No form intermediate.” The words near the foot of the structure—“Parent of Marsupials and Placentals”—indicate the subject about which he is thinking and drawing: the supposed common ancestry of marsupial and placental mammals. Starting from the foot of the treelike structure, three branchings fan out. This is the first time we see such a sprawling exuberance in Darwin’s drawings, and we seem to be looking at a new form: the leafless crown of a tree felled in winter.

Two of these branchings are labeled “marsupials” and “rodents”; the third remains anonymous. The term “rodents” placed in opposition to marsupials indicates that the issue here is placental rodents. On the right, a line connects the words “no form intermediate” with the trunk of the tree. In this way, Darwin apparently wanted to stress the absence of an intermediate species between rodents and marsupials. To the right, a line connects a circle drawn around a cluster of five points with the text, “If these had all given descendants then this wd have been a great series.” In the context of his sketch, this statement seems to be superfluous, for in the case where all these five “dots which represent Genera” had produced offspring, this would inevitably have resulted in a “great series” of descendant “dots which represent Genera,” connected by lines of affinity. Nonetheless, below it will become clear how not letting this happen in his sketch, due to lack of space, helped Darwin think about the conditions for development of genera.

Once more, Darwin’s process of drawing and writing focuses on a point of crucial importance for the plausibility of his theory—this time, common ancestry. Just as it was extremely hard to pinpoint the origin of species from preceding ones—the weak point we witnessed Darwin trying overcome in previous sections—so it was even more difficult to make a reasonable case for the plausibility of the basic idea of his theory—that is, that species have a common ancestor. But if he could show common ancestry of species—or of genera or even of orders—that would have been a much more convincing argument for his theory than the arguments we have seen him construct in the previous sections. Obviously, the main area in which he could have used convincing arguments derived from common ancestry is biological classification, which then and now is the field in which naturalists gather to exchange news and settle disputes. This is apparent on many pages in *Origin,* foremost, of course, in Chapter 13, devoted mainly to classification.

At the time Darwin drew this sketch, Robert Waterhouse was one of the greatest authorities in the field of classification. In 1843, Waterhouse entered the Department of Natural History of the British Museum and later became curator of the Department of Geology. He described the specimens of mammals and insects Darwin collected during his voyage on the *Beagle*. Waterhouse indicated to Darwin an important case of common evolutionary ancestry: the small South American rodent vizcacha that was assumed to be related to marsupials. Evidently this animal is the subject of the drawing: an ancestor of the vizcacha is supposed to be the “Parent of Marsupials and Placentals,” that is, the common ancestor of rodents and marsupials.

Knowing this context reframes understanding the abundant sprawling of the branches in the drawing: this is how Darwin depicts the common origin of an extensive order and an equally extensive *infraclass* (a subdivision of a subclass of the class of mammals). The order of rodents comprises a huge diversity of mammalian species, while marsupials all belong to an equally diverse infraclass. Furthermore, both the order and the infraclass are connected to one another in a complex way.

In *Origin,* two years after sketching the drawing examined here, Darwin lifted a corner of the veil covering part of the intricacies:

Mr. Waterhouse has remarked that, when a member belonging to one group of animals exhibits an affinity to a quite distinct group, this affinity in most cases is general and not special: thus, according to Mr. Waterhouse, of all Rodents, the bizcacha [i.e., vizcacha] is most nearly related to Marsupials; but in the points in which it approaches this order, its relations are general, and not to any one marsupial species more than to another. As the points of affinity of the bizcacha to Marsupials are believed to be real and not merely adaptive, they are due on my theory to inheritance in common. Therefore we must suppose either that all Rodents, including the bizcacha, branched off from some very ancient Marsupial, which will have had a character in some degree intermediate with respect to all existing Marsupials; or that both Rodents and Marsupials branched off from a common progenitor, and that both groups have since undergone much modification in divergent directions. (Darwin 1859, p. 430)

Darwin leaves unanswered the question of whether the ancestor was a marsupial or belonged to an undefined taxon. But it is reasonable to assume that, two years earlier, the drawing had helped him to consider the equal plausibility of both alternatives.

It is obvious for another reason, too, that Darwin was not doodling just for pleasure. The evidence is the text referring to the five encircled points: “If these had all given descendants then this wd have been a great series.” We are witnessing here one of the moments in which Darwin was sharpening his ideas about phylogeny—that is, how evolution simultaneously shapes both nature and its taxonomy and forces some species in the direction of extinction, thereby banishing them to the fossil regions of the taxonomy. A result of his musings appears in *Origin* (probably not by accident) almost immediately before the appearance of the common ancestor of rodents and marsupials. Here is his explanation of why nature prefers to converge to extensive, dominant species and to higher taxa:

As the modified descendants of dominant species, belonging to the larger genera, tend to inherit the advantages, which made the groups to which they belong large and their parents dominant, they are almost sure to spread widely, and to seize on more and more places in the economy of nature. The larger and more dominant groups thus tend to go on increasing in size; and they consequently supplant many smaller and feebler groups. Thus we can account for the fact that all organisms, recent and extinct, are included under a few great orders, under still fewer classes, and all in one great natural system. (Darwin 1859, pp. 428–429)

It becomes clear now what the five encircled points represent: one of the countless smaller and weaker taxa that, according to Darwin’s description, have been crowded out by more dominant ones due to lack of space. This small part of the sketch illustrates the proof by contradiction in the text, showing how placental mammal and marsupial species were successful, and reached the pages of *Origin,* while weaker ones were doomed to become anonymous points on an equally anonymous sheet of paper.

This “Let Dots Represent Genera” sketch once more indicates the versatility of the explanatory power of Darwin’s sketches. The first sketches helped him to clarify the equilibrium between speciation as a cause of adaptation to external conditions, on the one hand, and extinction explained by monadism, on the other. The “I think” sketch helped him to understand the dynamic balance between speciation and extinction, but also the evolutionary genealogical distance between descendants of a common ancestor. The “endless piece of seaweed dividing” showed how, despite a lack of boundaries between species, speciation is imaginable by means of the concept of aversion in nature to interracial crossing, but also how species “may for a long period continue transmitting unaltered descendants.” The barnacle sketch helped Darwin to imagine the fragmentation and radiation of families into genera, into species, and into varieties, as well as to suspect (but not to find) the underlying universal law. The entangled mass of sketches in the previous section depict phylogenetic mappings of embryonic forms. Finally, the “Let Dots Represent Genera” sketch helped him to argue common ancestry and to explain how smaller and weaker taxa have been superseded by more dominant ones, due to lack of space. These different manifestations of the explanatory power of the points, lines, and spaces of his sketches—as well as the sketches considered in the following sections—are each a step in the development of the multifunctionality of the diagram that, years later, would become an indispensable element of *Origin.*

## Lines and Points in Geological Space-Time (1857)

**Fig. 14:**
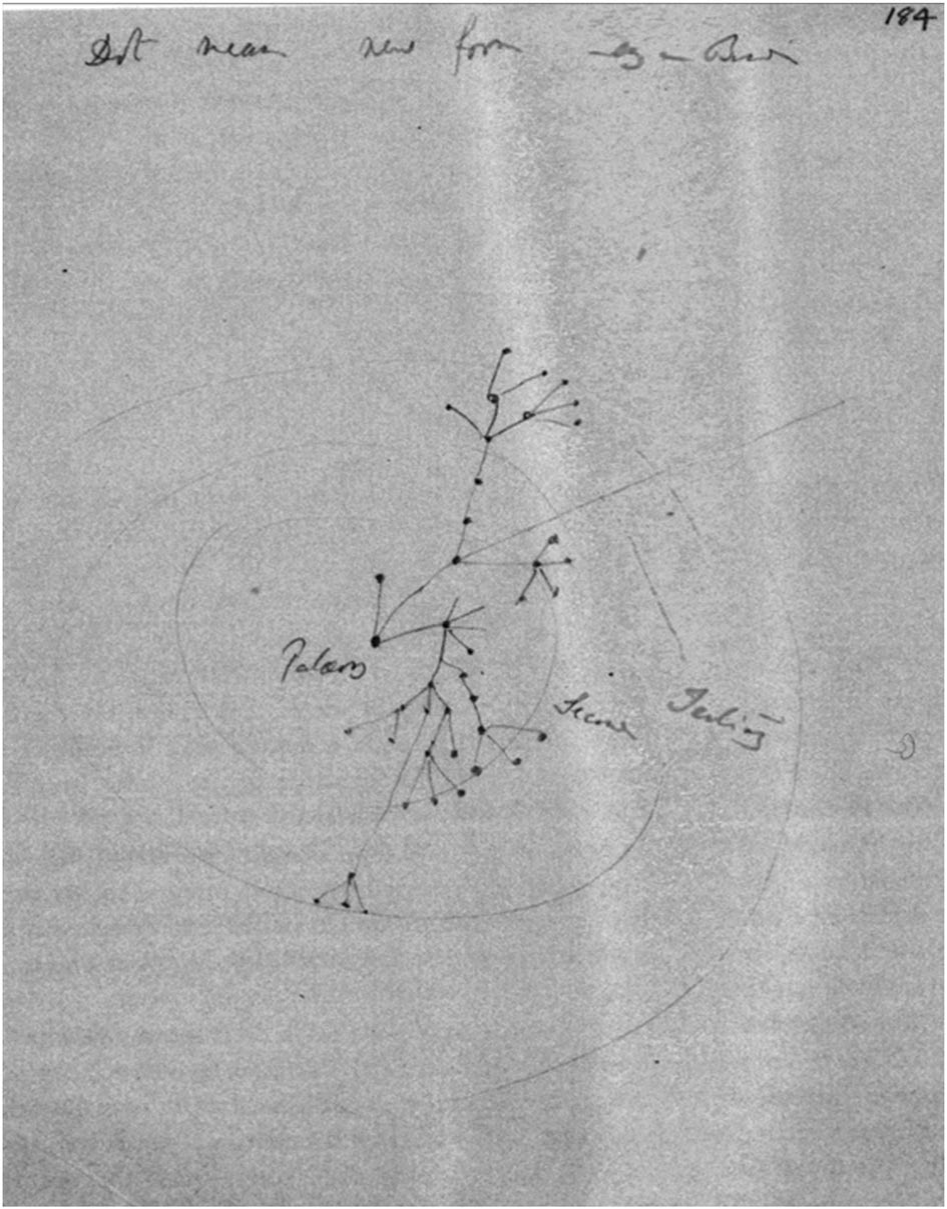
Sketch on a loose sheet, depicting evolutionary radiation in geological space-time (circa 1857), (Cambridge University Library MS.DAR.205.184r; reproduced by kind permission of the Syndics of Cambridge University Library.)

In the second sketch dating from 1857 or 1858, the geological time scale is represented by concentric circles (Fig. 14).

Archibald and Voss state that Darwin possibly copied the idea of depicting geologic time as a circle from diagrams published by Louis Agassiz in 1848 and Heinrich Bronn between 1850 and 1856 (Archibald 2014, p. 89; Voss 2010, p. 108). But these authors overlook the fact that the “I think” sketch (as we saw in “I think…” (1837) section), drawn by Darwin many years earlier in 1837, also has a centrifugal notion of time, as time elapses outward along its branches as they grow in all directions. In any case, in the second sketch of 1857, Darwin made the centrifugal course of time explicit by drawing concentric circles and placing the origin of the figure in its spatiotemporal center.

At the top of the sheet, Darwin wrote the caption, “Dot means new form,” followed by two or three illegible words^14^ Once more, the elements of the drawing are lines and points, but now a new shape appears as well: three more or less complete concentric circles and, between the outer and the middle circles, two arcs of a circle. In the common center of the circles, a point-line structure begins to grow).^15^ It seems similar to earlier arrangements, but there is one remarkable difference: the offshoots furcate in two diametrically opposed directions. One of the lines continues beyond the outer circumference. In the circles, from the inside out, are written the words “Paleoz,” “Second,” and “Tertiary,” referring to geological periods. An intriguing feature of the drawing, however, is the fact that it produces the sensation of optical depth: on a flat surface, Darwin suggests a concave shape centered around the extreme low point “Paleoz.” Although it was certainly not Darwin’s intention to create an optical illusion, one seems to perceive the depth of the drawing after a moment’s reflection, in much the same way as when one enjoys one of M. C. Escher’s prints.

In the meantime, the meaning of the drawing and the related text has become clarified. The circles symbolize three geologic eras, but also three layers in the geological stratigraphy. Following Charles Lyell’s naming system—the Paleozoic, the more recent Secondary (today called the Mesozoic), and the still younger Tertiary—Darwin sketched a centrifugal, geologic sequence of time. But the time lapse is not merely centrifugal; it also flows upward from the extreme central depth of the concave space—the more distant from the center, the more recent any point is. We can now understand why the growth of the line-point structure starts in the center. It is also evident why both sides of the structure sprawl in two opposed directions: evolving taxa are striving for maximal distance from one another in the full 360 degrees of space-time on the geological scale, trying to evade the fate of extinction suffered by the five fatally endangered genera in the “Let Dots Represent Genera” sketch analyzed in “The Parent of Marsupials and Placentals” (1857) section.

The significance of the words “Dot means new form” also becomes evident: the points symbolize fossil species in geologic strata. In this way, Darwin ingeniously interconnected space and time. Beyond the outer circumference of the Tertiary, we find the Quaternary, the current geological period. The continuous line to the upper right does not contain any dot that “means new form.” It is obvious that this line relates to the lines in the diagram of *Origin*, which “may for a long period continue transmitting unaltered descendants” (Darwin 1859, p. 121).

In summary, the figure shows the origin of species from the earliest life form’s first moment until the present. What Darwin was thinking while making this sketch can best be described in his own words, written down in the famous “tree of life” allegory, the coda to the first part of his “long argument” in *Origin*. In this allegory, he summarized a number of his earlier diagrammatic exercises:

Of the many twigs which flourished when the tree was a mere bush, only two or three, now grown into great branches, yet survive and bear all the other branches; so with the species which lived during long-past geological periods, very few now have living and modified descendants. From the first growth of the tree, many a limb and branch has decayed and dropped off; and these lost branches of various sizes may represent those whole orders, families, and genera which have now no living representatives, which are known to us only from having been found in a fossil state. (Darwin 1859, p. 130)

This is exactly what the sketch analyzed here shows: extinction and fossilization. But, more precisely, the sketch, with its one branch to the Quaternary, also shows the imperfection of the geological record—which Darwin described in another passage in *Origin,* in which he states that extinction is the inevitable consequence of his theory. He also explained why, nevertheless, hardly any fossil evidence of extinction can be found:

But just in proportion as this process of extermination has acted on an enormous scale, so must the number of intermediate varieties, which have formerly existed on the earth, be truly enormous. Why then is not every geological formation and every stratum full of such intermediate links? Geology assuredly does not reveal any such finely graduated organic chain; and this, perhaps, is the most obvious and gravest objection which can be urged against my theory. The explanation lies, as I believe, in the extreme imperfection of the geological record. (Darwin 1859, p. 280)

Heather Brink-Roby sees a potential contradiction in one branch of the sketch, which grows back against the centrifugal sequence of time (indicated by the blue arrow in Fig. 16). Trying to answer the question of whether Darwin was able successfully to represent evolving nature within the limits of his visual means (two spatial dimensions on the flat surface of his paper in combination with text), Brink-Roby concludes that these means appear to be insufficient. She claims that Darwin, when trying to show the morphological divergence of species in all directions by pairs of morphological coordinates, needed two spatial dimensions and therefore had to sacrifice the dimension of time. However, she concluded: “The very fact that both dimensions are occupied by morphology makes time invisible” (Brink-Roby 2009, pp. 256–260). My interpretation differs from hers. In the drawing, time is not invisible at all. On the contrary, it is prominently present in Darwin’s centrifugal depiction of circular, geological periods.

An additional reason why it is unimaginable that time is absent in this sketch is the fact that Darwin transferred the sketch and what it clarified for him—the incompleteness of the geological record—to the diagram in *Origin*. To do that, he had to bend it upward by transferring the lapse of time to the vertical axis of the diagram. Had time been absent in the sketch, as Brink-Roby contends, this transfer would have been impossible. Consequently, I agree with Bredekamp, who concluded that, at the place marked by the blue arrow, we see Darwin merely committing a *lapsus pennae*, a slip of the pen (Bredekamp 2006, p. 38).

## Three Diagrams for the “Big Species Book” (1858–1859)

When making the drawing discussed in the previous section, Darwin was also busy writing a comprehensive book about his theory, which he called his “Big Species Book.” What he was doing was known only to a very small circle of friends. As is widely known, during a visit to Down House in 1856, Lyell, having browsed through the manuscript, urged Darwin to prepare it for publication.^16^ Instead, Darwin continued working on it (see van Wyhe 2007). In June 1858—having finished more than seven hundred pages, about two-thirds of the estimated size of the book—he was alarmed by receiving a letter from Alfred Russel Wallace, who was at the time doing extensive research in Southeast Asia. Wallace had come to the same conclusion that Darwin had been preparing all these years: namely, that natural selection must be the mechanism that causes evolution in nature.^17^ After the Linnean Society’s famous joint publication of Darwin’s and Wallace’s papers (1858), Darwin started working frantically on a condensed version of the “Big Species Book,” which appeared in the fall of 1859 under the title *On the Origin of Species*. In 1975, the original text of the “Big Species Book” was published under the title *Charles Darwin’s Natural Selection: Being the Second Part of His Big Species Book, Written from 1856 to 1858* (Darwin 1975; see also Voss 2010, pp. 114–115; Archibald 2014, p. 95).

**Fig. 15:**
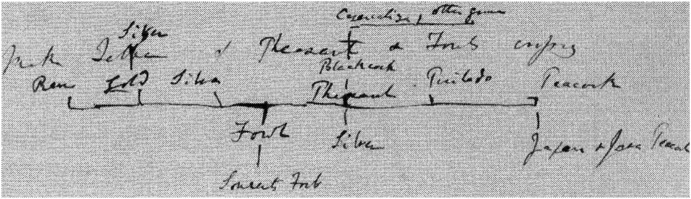
“Table of Pheasant & Fowls Crossing” for the “Big Species Book.” (Cambridge University Library MS.DAR.205.7[1].86r; reproduced by kind permission of the Syndics of Cambridge University Library.)

**Fig. 16:**
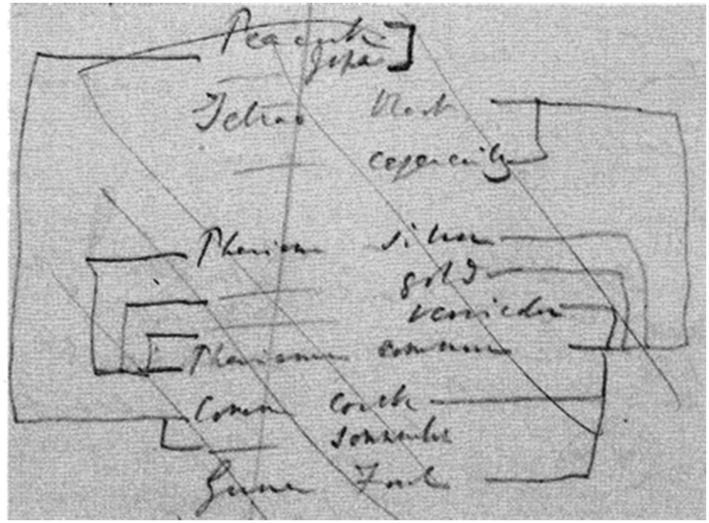
A scheme, which Darwin rejected for publication in the “Big Species Book,” showing brackets of pairs of birds known to hybridize. (Cambridge University Library MS.DAR.205.7[1].33v; reproduced by kind permission of the Syndics of Cambridge University Library.)

**Fig. 17:**
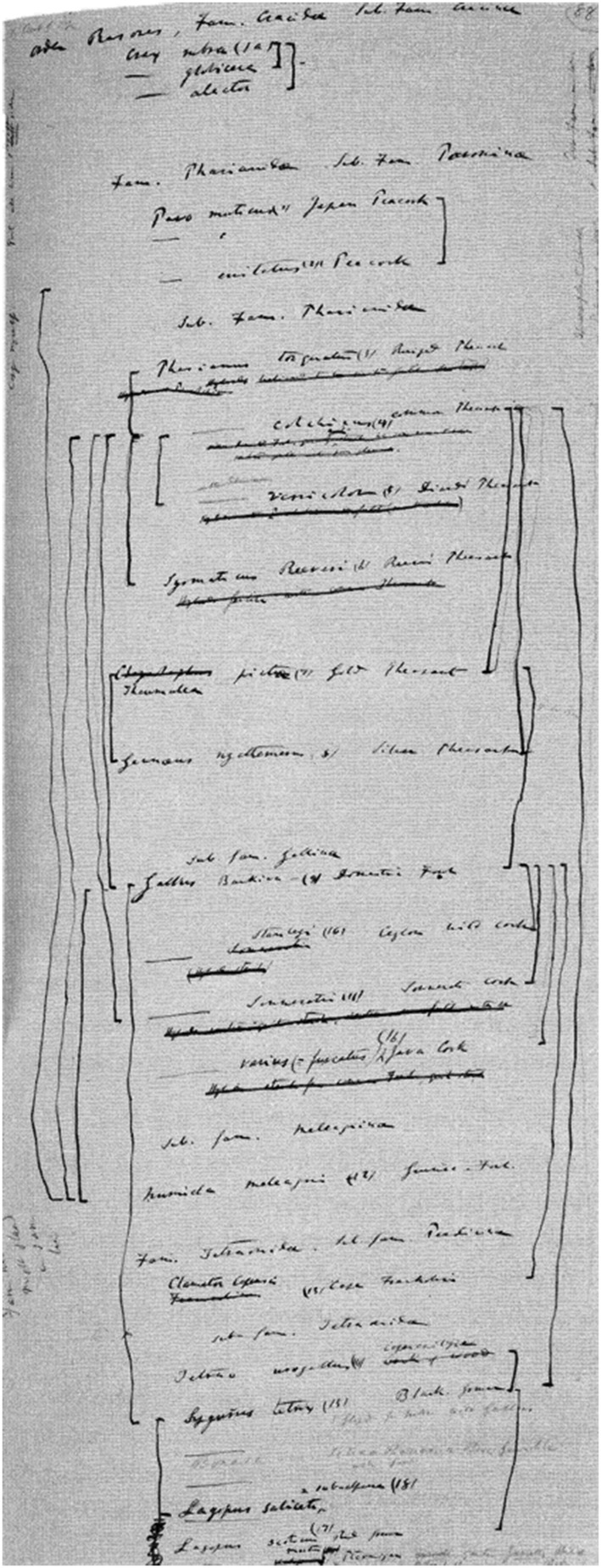
A diagram for the “Big Species Book” showing brackets of pairs of birds known to hybridize. (Cambridge University Library MS.DAR.12.88: reproduced by kind permission of the Syndics of Cambridge University Library.)

This book contains two hybrid diagrams (see Figs. 15 and 16), as well as a two-page precursor of the diagram in *Origin* (Fig. 17), which will be discussed in “The Centerfold of *Natural Selection* (1858)” section. It should be noted that, given Darwin’s intention to publish the book, the status of these sketched diagrams differs from that of the previous sketches, which had a heuristic function. In the “Big Species Book,” the diagrams were meant to play a didactic role, helping the reader grasp the theory more easily. It cannot be ruled out that these diagrams also helped Darwin’s thinking process, but their primary role was a didactic one. In this sense, these three diagrams were a kind of pilot study for the summative didactic diagram included in *Origin* depicting the crucial aspects of his theory, which drawing his various sketches had helped him to uncover.

One of the diagrams, which is the most similar to the ones I have analyzed in the previous sections, carries the title—or rather an instruction Darwin addressed to himself—“Make Table of Pheasant & Fowls Crossing” (Fig. 15). The result is a so-called *unrooted tree*, a term used today by biologists to indicate phylogenetic schemes in which a common ancestor is absent (Archibald 2014, p. 96). Behind the words “Pheasant & Fowls” are hidden dizzying taxonomic networks: that of the Phasianinae (a subfamily of the Phasianidae family, which belongs to the Galliformes order) and that of the Galloanserae superorder. In this diagram, Darwin summarizes whether organisms belonging to these taxa are capable of crossing with those belonging to others.

The shapes of the other diagrams (Figs. 16 and 17) are dissimilar. They appear to be schemes in which Darwin brackets pairs of birds known to hybridize. In Fig. 17, Darwin places various genera of the order Rasores (e.g., pheasants and peacocks) above and below each other, using brackets on either side to indicate species able to hybridize: “The brackets imply that hybrid offspring has been produced by the two forms so connected” (Darwin 1975, p. 436). Barely legible numbers next to the names of the species refer to several pages of footnotes indicating the degree of fertility of the offspring of the crossed birds (see Archibald 2014, p. 95). Figure 16 shows a rejected attempt to do the same.

**Fig. 18:**
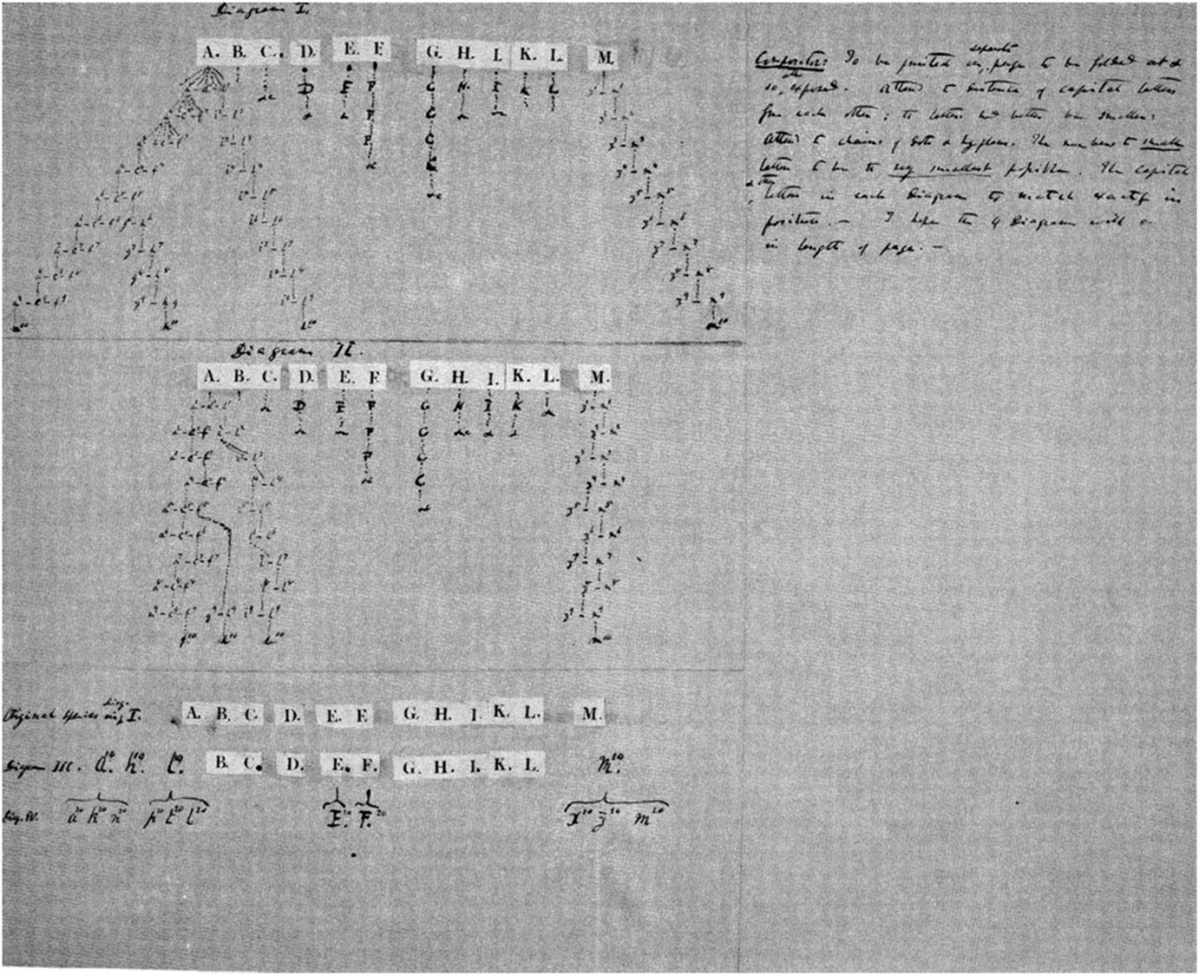
Four diagrams included in the “Big Species Book,” showing the evolutionary results of different environmental conditions. (Cambridge University Library MS.DAR.10.2.26R-S; reproduced by kind permission of the Syndics of Cambridge University Library.)

Darwin is engaged here in summarizing results of research on hybridism, today called hybridization. He suspects that hybridism plays an important role in the origination of new species. But in order to substantiate his suspicions, he must first demonstrate that, in contrast to Georg-Louis Leclerc Buffon’s definition, hybridization can produce stable offspring (Farber 1972, pp. 262–263). Darwin does not agree with Buffon; he believes—as we previously noted when inspecting the seaweed sketch—that organisms in nature have an aversion to interracial crossing that can be suppressed by breeders. He also believes that when organisms in nature do overcome their aversion to interbreeding, the offspring may eventually have evolutionary advantages. But that can be the case only if the offspring are fertile, and that is what he wants to demonstrate here, as is evident from a description of these diagrams in the “Big Species Book.” There, he states that in the diagrams, the reader is seeing bastards of “all the well authenticated crosses which I have heard of in one order of Bird, the Rasores; in order that those who have not attended to the subject, may know how numerous the crosses have been & between what different forms” (Darwin 1975, p. 434). The sole purpose of the big diagram in Fig. 18 appears to be to summarize the results of his empirical investigation of hybridism in publishable form in his “Big Species Book.”

## The Centerfold of *Natural Selection* (1858)

“The complex action of these several principles, namely, natural selection, divergence & extinction, may be best, yet imperfectly illustrated by the following Diagram, printed on a folded sheet for convenience of reference. This diagram will show the manner, in which I believe species descend from each other & therefore shall be explained in detail: it will, also, clearly show several points of doubts & difficulty” (Darwin 1975, pp. 238–239). Thus begins the explanation of four diagrams Darwin intended to include in his “Big Species Book” (Fig. 18). The diagrams, numbered I through IV, are a fourfold predecessor of the unified diagram in *Origin*. There exist, however, remarkable differences between them.

The upper right corner contains a carefully worded instruction about typographic spaces; the typesetter had to leave more space between the capital letters C and D, D and E, F and G, and L and M than between the others. It is clear why: in Darwin’s diagram in *Origin*, horizontal distances symbolize the measure of divergence, and that is true here too.

Darwin’s description of the four diagrams of the “Big Species Book” runs to about 3500 words, or 20 percent more than the 2700 words he needed for the legenda of the diagram in *Origin* (Archibald 2014, pp. 96–97). All four diagrams represent the evolutionary development of the same series of plant species under various conditions in “a continuous area, not separated by borders.” In Diagram I, A through M represent plant species within a genus, varying from left to right in their need for water, “A the most moisture loving & M the least moisture-loving species.” From A, three evolutionary developments start branching top down, eventually resulting in three species; and from M, one evolutionary line begins to grow. The lines emanating from B through L seem to become extinct altogether, but this impression is incorrect, for at the terminus of these lines Darwin writes “&c.” These species “are supposed to have transmitted unaltered descendents (sic)” (Darwin 1975, p. 244).

In Diagram II, according to Archibald, “everything is the same as in diagram I … except that it is left to mere chance whether the more or less moisture-loving species are preserved” (2014, p. 100). This eventually results in a shorter morphological distance between the descendants on the horizontal axis, which means less divergence in characteristics. Ergo, natural selection causes a greater disparity in characteristics than chance alone, an idea that, according to Archibald, finds support in modern population studies (2014, p. 100).

Diagrams III and IV merely show the results of different parameters. Diagram III is the outcome after a much longer period of time than presented in II: the resulting morphological distance between L and M is longer, and in IV, species A and M have merged, after convergence, with their nearest neighbors.

According to Archibald, the diagrams intended for the “Big Species Book” are similar to the one in *Origin* (2014, p. 96). That depends, however, on what one means by similarity. Under a different interpretation, one may perceive a great dissimilarity between the two. There is, to begin with, a difference in abstraction, for diagrams I through IV show the result of a concrete situation in nature—the evolutionary outcome of the measure by which plants are “moisture loving”—while the diagram in *Origin* shows in an abstract way how species, genera, and families evolve.

Archibald also states that Darwin used the diagrams to carry out a thought experiment (2014, p. 99). I would instead propose that Darwin is conducting *four* thought experiments, wherein he imagines the above-mentioned “continuous area, not separated by borders” and poses for each experiment a what if question of the type, “What would we observe with regard to the evolution of species A through M if the different conditions mentioned above are specified?”.

Comparing these diagrams with the diagram in *Origin,* it emerges that the latter is more transparent: the lapse of time, turned upside down, is more visible on the vertical axis, as are the elements point, line, and space. Moreover, as opposed to the elements of the diagrams for the “Big Species Book,” the points, lines, and spaces in the diagram for *Origin* are initially neutral. They are blanks until the moment when Darwin assigns to them the then-relevant meaning needed to show readers certain aspects of how species, genera, and families evolve. The next section will show how this versatility of the points and lines in the sketches translates into the unsurpassed multifunctionality of the diagram.

## Conclusion: Evident and Hidden Heuristics in Darwin’s Sketches and Diagrams

In the “A Multifunctional Diagram (1859)” section, I concluded that Darwin assigned specific meanings to the graphic elements point, line, letter, numeral, and space in the diagram of *Origin*. In the subsequent sections, we found him developing and testing these meanings. The present section will show the ways in which the diagram of is the result of these exercises.

What is immediately noticeable in the diagram of *Origin* is the proliferation of branchings and straight lines. In fact, there is no place that is free of furcations, which are a legacy of the branching in all the sketches that have been examined in this article (with the exception of the seaweed sketch). The same is true for the linearity (foremost in the lines E and F), the heritage of the endless branches, which had been derived from the seaweed sketch and from the continuous line in the geological sketch.

Initially this seems tautological: branching diagrams show branching evolution and linear ones show linearity in evolutionary descent. But it is precisely this tautological aspect that Darwin needs. In “Introduction: Seventeen Sketches and a Diagram” section, I illustrated how Darwin appeared to be convinced of “the extraordinary difficulty which naturalists have experienced in describing, without the aid of a diagram, the various affinities which they perceive between the many living and extinct members of the same great natural class” (Darwin 1859, p. 431). This means that, according to his heuristics, a sketch and its text could not exist without each other, and the same goes for his didactics. I agree, therefore, with Brink-Roby, who suggested that the analogy is also about the relation between the diagram and the language with which Darwin supplements what it represents (2009, p. 265). Darwin evidently needed to switch between diagrammatic images and words, between thinking in pictures and explaining the pictures, and vice versa.

When looking at the branchings in detail, one notices boldfaced points representing the speciation in the phylogenetic nodes, as in the *L. anatifera* sketch (Fig. 9) and in compliance with the maxim “Dot means new form” of the geological sketch (Fig. 14). There, the look of the small, upward diagonals of the diagram, and thus their meaning, differs clearly from those of the sketches. They are all dotted and stand either for evolutionary development or for species that “may for a long period continue transmitting unaltered descendants.” They are a graphic inheritance of the seaweed sketch, the effect of whose idiosyncratic modus of “dividing,” is that the genus of the stem itself continues “unaltered.” The phenomenon of the separating secondary branch becoming a different one did not reach the diagram. By contrast, dotted lines like those in the first sketches, which indicated fossils alternating with missing links, are not found in the diagram. Interrupted lines in the diagram function as the earlier uninterrupted ones, obviously for typographic reasons.

The blank space as a synchronous expression of morphological distance in the geological sketch in Fig. 15 is also clearly visible. As shown in “I think …” (1837) section, the use of the element space in this way was implicitly present in the “I think” sketch. Explicitly, however, space entered the stage relatively late, in the circles in the centrifugal elapse of time in the geological sketch, but thereafter it remained expressly present. After his suboptimal attempts in the four diagrams drafted for the “Big Species Book,” Darwin projected the concave shape of the geological sketch onto the flat surface of his diagram in *Origin,* thus assigning the space to the horizontal lines and the passage of time to the vertical axis.

One more heuristic and didactic yield from the sketches found its way to the diagram of *Origin*: extinction. This aspect of the diagram, however, is not directly related to a meaning given by Darwin to graphical elements. Indeed, this appearance is indirect and thus contradictory. For although Figs. 13 and 14 show that the result of extinction—fossils or their absence (that is, missing links)—could be made patently visible, by contrast it is difficult to imagine how to visualize the very process by which living species are made to disappear. And yet, this is what the diagram does.

Recall that in the first two sketches (see Figs. 2, 3 and 4), fossils “appear like circles”—that is, points—alternating with spaces, which indicate places where links are missing in the line of development that would otherwise be uninterrupted. This way of representing extinction does not appear in the diagram, and it is evident that its supposed cause—monadism—cannot be shown in a drawing. By contrast, the “I think” sketch (Fig. 5) does show how the dynamic balance between speciation and extinction worked. To illustrate this, Darwin only had to create dead ends, by omitting crossbars at around half the extremities of the sketch. This method, which worked sub-optimally, as we have seen, did not make its way into the diagram of *Origin*.

The way in which Darwin represented the process of extinction in his “Let Dots Represent Genera” sketch (Fig. 14), however, left a demonstrable trace in the diagram. The five encircled points, which would have produced a “great series” of descendant “dots which represent Genera,” had there been enough space, are a proof by contradiction in the form of a sketch that displays how placental mammal and marsupial species were successful, while the five weaker ones became extinct. In the same way, Darwin has species dying out in the diagram. Having followed the development of species A through F until the 14,000^th^ generation, he focuses on the intermediate species, or “the other nine species (marked by capital letters) of our original genus,” stating that they “may for a long period continue transmitting unaltered descendants; and this is shown in the diagram by the dotted lines not prolonged far upwards from want of space” (Darwin 1859, p. 121). This means that initially these species are expected to continue unaltered. Lacking space, however, the diagram cannot show that, so Darwin discontinues the lines, in the same way in which in Fig. 11 he discontinued the “great series” of descendant “dots which represent Genera,” which would have been produced by the five encircled species, thus declaring them extinct. He illustrates what he is doing as follows: “But during the process of modification, represented in the diagram, another of our principles, namely that of extinction, will have played an important part…. It seems, therefore, to me extremely probable that they [i.e., the descendants of A and I] will have taken the places of, and thus exterminated, not only their parents (A) and (I), but likewise some of the original species which were most nearly related to their parents” (Darwin 1859, p. 121). Thus, we may conclude that all of Darwin’s sketches, except the embryological ones, left their traces on the final diagram.

## Footnotes

1 Page references are to the 2009 reprint edition. In fact, two more sketches made by Darwin exist, but these fall outside the scope of this article as they were drawn after the publication of *Origin* and thus did not contribute to its diagram. The sketches that will be discussed in this article are part of the *Charles Darwin Papers*, Manuscripts and University Archives, Cambridge University Library, https://www.lib.cam.ac.uk/collections/departments/manuscripts-university-archives/significant-archival-collections/darwin. Darwin’s Evolution Manuscripts are being published online at the *Cambridge Digital Library* (https://cudl.lib.cam.ac.uk/collections/darwin_mss/1) and simultaneously by the *Darwin Manuscripts Project* (https://www.amnh.org/research/darwin-manuscripts) in collaboration with the Charles Darwin Correspondence Project. Without these projects, of inestimable value for historical research and scholarship, the present article would not have been possible.

2 Voss, Bredekamp and Brink-Roby focus specifically on Darwin’s sketches in relation to the diagram in *Origin*. The same goes for Archibald’s chapter on Darwin’s sketches and diagram, although it is part of an overview of trees of life. Although in other comparable overviews, Darwin’s images are given attention, they are not treated similarly in their mutual cohesion; see Pietsch (2012) and Smith (2006), who positions Darwin’s diagram within contemporary visual culture. The works by Voss, Bredekamp, Brink-Roby, Archibald, as well as the present article, can be considered a particular niche between these extreme approaches in the literature.

3 I will omit a tree-like arrangement of birds, which was produced in 1856 by Alfred Russel Wallace (the co-founder of the theory of evolution by natural selection), that is distinct from visualizing evolutionary theories because it was not meant to convey phylogenetic information but rather address an issue in classification (Tassy 2010, p. 90).

4 Cambridge University Library MS.DAR.205.5.90r–v. Transcription by Archibald (2014, p. 85). Archibald states that the text between guillemets was added later by Darwin.

5 Cambridge University Library MS.DAR.205.5.90r–v. Transcription by J. David Archibald (2014, p. 85).

6 To Syms Covington, 30 March [1849], Darwin Correspondence Project, “Letter no. 1237,” accessed 17 August 2015, http://www.darwinproject.ac.uk/DCP-LETT-1237. Also published in *The Correspondence of Charles Darwin*, vol. 4.

7 To J. D. Hooker, 12 October [1849], Darwin Correspondence Project, “Letter no. 1260,” accessed 17 August 2015, http://www.darwinproject.ac.uk/DCP-LETT-1260. Also published in *The Correspondence of Charles Darwin*, vol. 4.

8 To G. R. Waterhouse, 26 July [1843], Darwin Correspondence Project, “Letter no. 684,” accessed 17 August 2015, http://www.darwinproject.ac.uk/DCP-LETT-684. Also published in *The Correspondence of Charles Darwin*, vol. 2.

9 Cambridge University Library MS.DAR.205.5127r–v. Transcription by J. David Archibald (2014, p. 86).

10 See Ospovat (1976) for Darwin’s ideas on recapitulation. Ospovat presented a convincing case that Darwin was not a follower of any theory of linear recapitulation, such as that of Haeckel. By contrast, he was a follower of von Baer, who agreed that there were different grades of development, but denied that they formed a single series (pp. 5–6). See also Abzhanov (2013).

11 Cambridge University Library MS.DAR.205.6.51r. Transcription by Archibald (2014, pp. 93–95).

12 https://www.biodiversitylibrary.org/item/106351#page/95/mode/1up. Accessed 14 May 2017. Secord erroneously states that Darwin penned his “no” in the margin of Dixon’s “Domestic Fowl and Ornamental Poultry.”

13 Cambridge University Library MS.DAR.205.5.183r. Transcription by Archibald (2014, p. 92).

14 Archibald (2014, p. 87) believes that the last word could be *bird*.

15 Cambridge University Library MS.DAR.205.184r.

16 To C. R. Darwin, 1-2 May [1856], Darwin Correspondence Project, “Letter no. 1862,” accessed on 15 November 2019, https://www.darwinproject.ac.uk/letter/DCP-LETT-1862.xml. Also published in *The Correspondence of Charles Darwin*, vol. 6.

17 To C. R. Darwin, 27 September [1857], Darwin Correspondence Project, “Letter no. 2145,” accessed on 17 August 2018, http://www.darwinproject.ac.uk/DCP-LETT-2145. Also published in *The Correspondence of Charles Darwin*, vol. 6.
